# SECTOR: structural entropy-based learning of spatiotemporal organisation in spatial transcriptomics

**DOI:** 10.1093/bioinformatics/btag367

**Published:** 2026-06-15

**Authors:** Li Huang, Jingyun Zhang, Weikang Gong, Guangjie Zeng, Hao Peng, Dongsheng Chen

**Affiliations:** State Key Laboratory of Common Mechanism Research for Major Diseases, Suzhou Institute of Systems Medicine, Chinese Academy of Medical Sciences and Peking Union Medical College, Suzhou, 215123, China; School of Cyber Science and Technology, Beihang University, Beijing, 100191, China; State Key Laboratory of Common Mechanism Research for Major Diseases, Suzhou Institute of Systems Medicine, Chinese Academy of Medical Sciences and Peking Union Medical College, Suzhou, 215123, China; School of Computer Science and Engineering, Beihang University, Beijing, 100191, China; School of Cyber Science and Technology, Beihang University, Beijing, 100191, China; State Key Laboratory of Common Mechanism Research for Major Diseases, Suzhou Institute of Systems Medicine, Chinese Academy of Medical Sciences and Peking Union Medical College, Suzhou, 215123, China

## Abstract

**Motivation:**

Spatial transcriptomics (ST) profiles gene expression in tissue context, enabling spatial domain detection. However, relatively few methods jointly recover discrete spatial domains and continuous within-section pseudotemporal trends in a single framework. Current spatiotemporal approaches often emphasise trajectory continuity to recover smooth progression-associated gradients, but this may blur neighbouring domain boundaries and reduce clustering accuracy. Conversely, specialised spatial clustering algorithms typically rely on external single-cell trajectory tools rather than providing an integrated, spatially aware pseudotime model.

**Results:**

We introduce SECTOR (Structural Entropy-based Clustering and pseudoTime ORdering), a lightweight deep graph learning framework that unifies spatial domain detection and pseudotime inference. SECTOR optimises a differentiable structural entropy (SE) objective on a fused spatial–expression graph, with spatial total variation regularisation to promote tissue continuity. Across seven benchmark datasets spanning standard and modern high-resolution ST platforms, SECTOR consistently outperformed existing spatiotemporal methods in clustering accuracy and matched or exceeded leading spatial clustering algorithms, while maintaining modest computational demands. In human breast cancer and mouse olfactory bulb case studies, SECTOR recovered spatially organised pseudotime patterns supported by semivariance, transition-gene, enrichment and marker-gene analyses. Together, these results show that SE-based learning provides an effective and scalable strategy for modelling within-section spatiotemporal organisation in ST.

**Availability:**

SECTOR is available on GitHub at https://github.com/lhbcb/SECTOR and archived on Figshare at https://doi.org/10.6084/m9.figshare.32029830.

## 1 Introduction

Single-cell transcriptomics has transformed our understanding of cellular heterogeneity, yet most assays lack explicit information about the spatial and temporal dimensions of biological organisation (Heitz *et al.* 2025). Spatial transcriptomics (ST) addresses the former by measuring gene expression together with tissue coordinates, enabling the construction of spatial cell atlases across sequencing- and imaging-based technologies. A central task in ST analysis is spatial domain identification (Fang *et al.* 2023, Yuan *et al.* 2024), which groups neighbouring spatial locations into molecularly coherent anatomical, functional or pathological regions. However, many biological processes such as development (Mantri *et al.* 2021) and disease progression (Smelik *et al.* 2025) vary continuously across tissue space. Consequently, there is a need to recover both discrete spatial domains and continuous progression-associated patterns. Consistent with common usage in trajectory inference, pseudotime denotes a latent ordering inferred from a static spatial section, rather than directly observed chronological time in cross-time-point samples.

Existing methods for spatial domain identification differ in their underlying assumptions and algorithms. Bayesian methods such as BayesSpace (Zhao *et al.* 2021) and BASS (Li and Zhou, 2022) impose probabilistic spatial priors to favour locally coherent domains. Deep graph-based learning methods such as STAGATE (Dong and Zhang, 2022) and GraphST (Long *et al.* 2023) integrate transcriptomic similarity with spatial adjacency to learn spatially aware embeddings. Context- or niche-aware methods, including CellCharter (Varrone *et al.* 2024), MENDER (Yuan, 2024), BANKSY (Singhal *et al.* 2024), and NicheCompass (Birk *et al.* 2025), model tissue structure using both spot/cell-level expression and local neighbourhood context. Other approaches treat domain detection as image-like segmentation, as in SpaSEG (Bai *et al.* 2025), or use scalable feature engineering followed by conventional clustering, as in NichePCA (Schaub *et al.*^2025^). Despite this variety, benchmark studies (Hu *et al.* 2024, Yuan *et al.* 2024) indicate that no single method performs optimally across all platforms and tissue contexts. Moreover, when temporal structure is of interest, these methods are typically combined post hoc with generic single-cell trajectory tools rather than providing an integrated, spatially aware pseudotime model.

Several graph-based spatiotemporal methods have been developed to incorporate spatial information into pseudotime inference. SpaceFlow (Ren *et al.* 2022) learns a spatially regularised embedding to derive a pseudo-spatiotemporal map. stLearn (Pham *et al.* 2023) combines gene expression, physical distance and optional morphology in a graph-based framework. CASCAT (Yu *et al.* 2025) uses a causally pruned spatial graph to infer a Markovian tree-shaped trajectory. stTrace (Song *et al.* 2025) implements an entropy-based graph optimisation framework, but requires a precomputed developmental level for each spot or cell. These approaches improve upon directly applying single-cell trajectory tools such as Slingshot (Street *et al.* 2018) and Monocle (Trapnell *et al.* 2014) to ST data. However, current spatiotemporal models can show lower clustering accuracy than state-of-the-art clustering-focused methods (Hu *et al.* 2024, Yuan *et al.* 2024), even though reliable domain recovery is important for robust pseudotime inference (Yu *et al.* 2025). This gap motivates a unified framework that jointly learns spatial domains and within-section pseudotime, while preserving clustering performance.

In this study, we present SECTOR (Structural Entropy-based Clustering and pseudoTime ORdering), a lightweight deep graph learning model that unifies spatial domain detection and pseudotime inference. Although structural entropy (SE) was originally defined on discrete graph partitions or partitioning trees (Li and Pan 2016), SECTOR reformulates it as a differentiable loss on a fused spatial–expression graph, modelled through a graph-encoding tree representing root–clusters–spatial locations. By minimising SE with spatial total variation regularisation, SECTOR favours low-entropy, spatially coherent domains while retaining the graph connectivity required for pseudotime reconstruction. Across diverse ST technologies, SECTOR achieves strong spatial domain detection performance, improves pseudotime ordering relative to existing spatiotemporal methods, and maintains competitive runtime and memory usage.

## 2 Materials and methods

### 2.1 Datasets

This study involves nine publicly available ST datasets spanning diverse sequencing-based and imaging-based technologies (Supplementary Section S1, available as supplementary data at *Bioinformatics* online). Seven datasets were used for spatial-clustering benchmarking and grouped by platform type and scale: sequencing-based ST, including 10x Visium human postmortem dorsolateral prefrontal cortex (DLPFC) (Maynard *et al.* 2021) and Stereo-seq mouse embryos (Chen *et al.* 2022); imaging-based ST, including MERFISH mouse hypothalamus (Moffitt *et al.* 2018), STARmap mouse cortex (Wang *et al.* 2018) and BaristaSeq mouse primary cortex (Long *et al.* 2023); and modern large-scale high-resolution ST, including Visium HD colorectal cancer (CRC) (Oliveira *et al.* 2025) and Xenium infiltrating ductal carcinoma (IDC) (Bhuva *et al.* 2024). Two additional datasets, 10x Visium HER2ST breast cancer (Andersson *et al.* 2021) and MERFISH mouse olfactory bulb (OB) (Zhang *et al.* 2023), were used as case studies for spatiotemporal inference and downstream analyses. The first five datasets were selected for their widespread use in spatial clustering benchmarking, Visium HD and Xenium were included to assess SECTOR on large-scale high-resolution platforms, and the two case-study datasets were chosen for their previous use in spatiotemporal analyses. Dataset details and preprocessing procedures are provided in Supplementary Section S1, available as supplementary data at *Bioinformatics* online.

### 2.2 Graph construction and fusion

**Figure 1:**
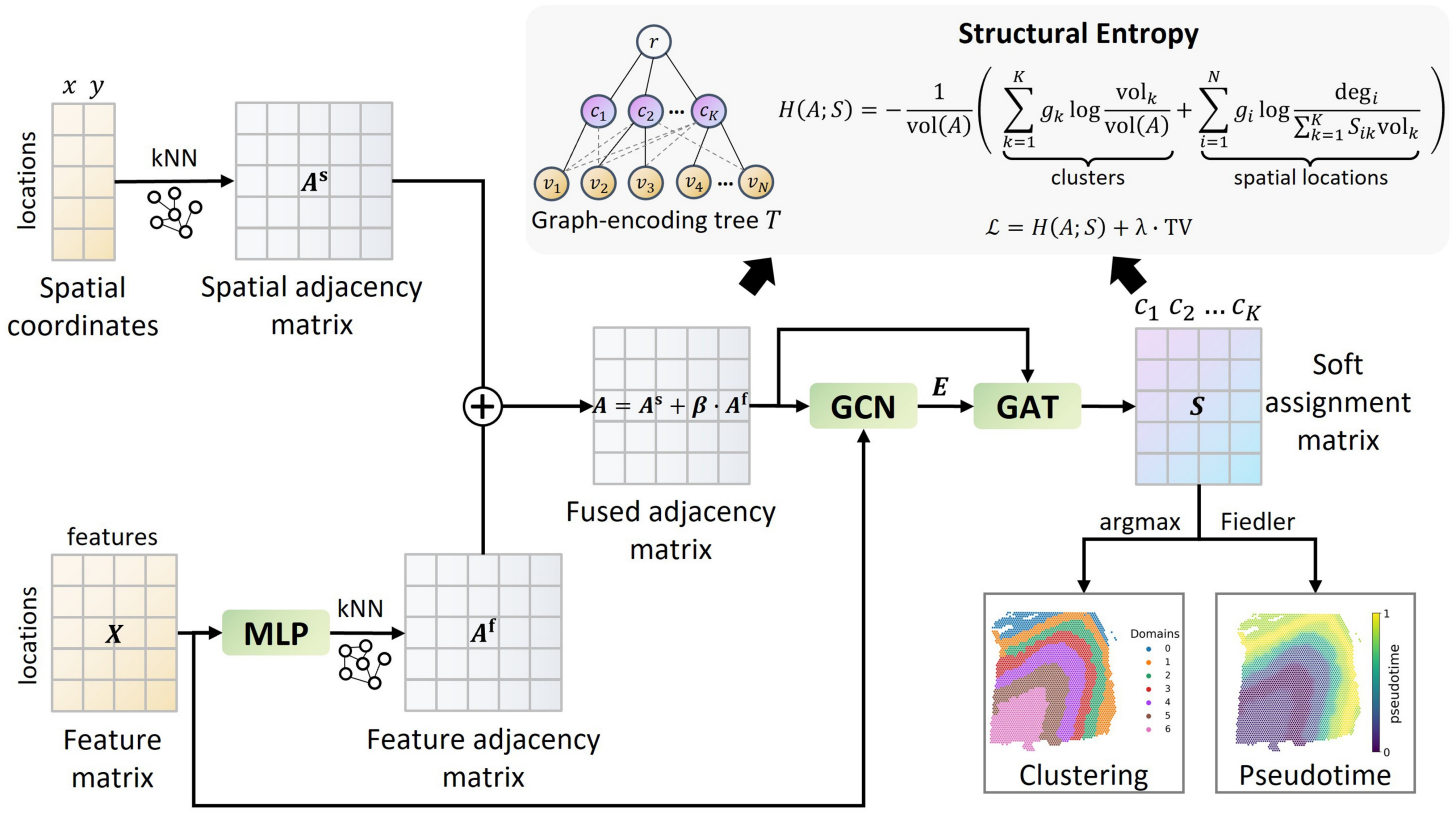
Overview of the SECTOR framework. SECTOR takes spatial coordinates and a PCA-based gene–expression feature matrix as input. It constructs a fused spatial–expression adjacency matrix *A* by combining a spatial adjacency matrix *A*^s^, derived from a *k*-nearest neighbour (kNN) graph on coordinates, and a feature adjacency matrix *A*^f^, derived from a kNN graph of a multilayer perceptron (MLP)-encoded feature embedding from the PCA feature matrix. A lightweight module, comprising a graph convolutional network (GCN)-style encoder and a graph attention (GAT)-based assignment head, learns a soft assignment matrix *S*. The model minimises a differentiable structural entropy (SE) objective *H*(*A*; *S*) on a two-layer graph-encoding tree (root–clusters–spatial locations), with spatial total variation (TV) regularisation to promote tissue continuity. SECTOR outputs discrete spatial domains by argmax of the learned assignments and continuous pseudotime from the Fiedler vector of the induced cluster-level graph. Here, spatial locations refer to spots, cells or bins depending on the ST platform.

For a dataset with *N* spatial locations, referring to spots, cells or bins depending on the ST platform, SECTOR uses spatial coordinates $\left(x_{i},y_{i}\right)$ for each location *i* and a *d*-dimensional principal component analysis (PCA) feature matrix $X\in R^{N\times d}$ computed from $N_{\text{HVG}}$ highly variable genes (HVGs). To capture both physical proximity and transcriptomic similarity, SECTOR constructs a fused spatial–expression graph *G* from two sparse weighted graphs (Fig. 1). The spatial graph with adjacency matrix $A^{s}\in R^{N\times N}$ is built as a symmetric *k*-nearest neighbour graph in coordinate space, with edge weights defined by a Euclidean-distance Gaussian kernel; $k_{\text{spatial}}$ controls the number of spatial neighbours retained for each location and is set to 6 by default. The feature graph with adjacency matrix $A^{f}\in R^{N\times N}$ is a symmetric kNN graph in an expression-embedding space generated by a multilayer perceptron (MLP) from *X*, with cosine-similarity edge weights; $k_{\text{feat}}$ controls the number of expression-space neighbours retained for each location and is set to 1 by default. The final fused graph *G* has adjacency matrix


(1) $$A=A^{s}+\beta A^{f},$$

Where β≥0 controls the contribution of the feature graph. This fused graph is used for subsequent SE-based deep graph learning. Full details of graph construction are provided in Supplementary Section S2, available as supplementary data at *Bioinformatics* online.

### 2.3 SE-based learning

#### 2.3.1 Classical SE

Structural entropy measures the minimum description length required to encode random walks on a graph (Li and Pan 2016). For the fused graph *G* with weighted adjacency *A* and total volume ${\text{vol}}_{G}$, the SE of *G* under a hierarchical partitioning tree *T* is


(2) $$H^{T}(G)=-\sum_{\alpha \in T\backslash \{r\}}\frac{g_{\alpha }}{{\text{vol}}_{G}}\text{log}\;\frac{{\text{vol}}_{\alpha }}{{\text{vol}}_{\alpha^{\text{parent}}}},$$

Where *r* is the root of the tree, ${\text{vol}}_{\alpha }$ is the volume (total degree) of node α, ${\text{vol}}_{\alpha^{\text{parent}}}$ is the volume of its parent, and $g_{\alpha }$ is the cut volume between α and the rest of the graph. Minimising *H^T^*(*G*) maximises within-module connectivity while minimising inter-module transitions.

#### 2.3.2 Differentiable SE with soft assignments

Classical SE optimisation is discrete and combinatorial. Zhang *et al.* (2025) reformulated SE as a differentiable objective for generic deep graph clustering, typically applied to binary graphs with stacked GNN layers and reconstruction losses. SECTOR instead introduces architectural adaptations tailored for ST, operating on the fused spatial–expression graph *G* with continuous edge weights and restricting the SE hierarchy to a two-layer graph-encoding tree (root–clusters–spatial locations). Specifically, SECTOR models spatial domains as a soft partition of *G*. Given the fused weighted adjacency matrix *A* with *N* nodes and a target of *K* domains, let $S\in R^{N\times K}$ denote a soft assignment matrix where *S_ik_* represents the probability that node *i* belongs to cluster *k*, subject to $\sum_{k=1}^{K}S_{ik}=1$. We employ a fixed two-layer encoding tree *T* consisting of a root *r*, *K* cluster nodes ($c_{1},\ldots ,c_{K}$), and *N* leaf nodes ($v_{1},\ldots ,v_{N}$) corresponding to individual spatial locations. Based on this structure, we derive a continuous relaxation of SE in terms of *S* and *A* (Fig. 1). The full derivation is provided in Supplementary Section S3, available as supplementary data at *Bioinformatics* online. Let $\deg_{i}$ denote the degree (volume) of node *i* and let vol(A) represent the total volume of *G*. For each cluster *k*, we define its soft volume and cut volume as ${\text{vol}}_{k}$ and $g_{k}$, respectively; similarly, we denote the node-level cut volume as $g_{i}$. All of these quantities are formulated as smooth functions of *S* and *A* (see Supplementary Section S3, available as supplementary data at *Bioinformatics* online for explicit expressions). The resulting two-layer differentiable SE is defined as


(3) $$H(A;S)=-\frac{1}{\text{vol}(A)}(\sum_{k=1}^{K}g_{k}\;\text{log}\;\frac{{\text{vol}}_{k}}{\text{vol}(A)}+\sum_{i=1}^{N}g_{i}\;\text{log}\;\frac{\deg_{i}}{\sum_{k=1}^{K}S_{ik}{\text{vol}}_{k}}).$$

Minimising *H*(*A*; *S*) discourages random walks from crossing cluster boundaries, thereby favouring domains that are internally cohesive and separated by small boundary volume.

#### 2.3.3 SECTOR architecture

Building on this differentiable SE formulation, SECTOR learns the soft assignment matrix *S* using a lightweight neural architecture (Fig. 1).

##### 2.3.3.1 MLP feature encoder

As described in Section 2.2, a two-layer MLP feature encoder with ReLU activation transforms the PCA feature matrix $X={\left[x_{1},\ldots ,x_{N}\right]}^{T}\in R^{N\times d}$ into a low-dimensional representation *E*^f^


(4) $$E^{f}=\phi_{\text{MLP}}(X)\in R^{N\times d_{\text{emb}}},$$

Which is used to construct the feature adjacency matrix *A*^f^ via kNN.

##### 2.3.3.2 Graph convolutional network (GCN) layer

A customised GCN-style graph encoder (Kipf and Welling 2017) aggregates *X* over the fused adjacency matrix *A* to produce context-aware embeddings *E*


(5) $$E=\phi_{\text{GCN}}(X,A)\in R^{N\times d_{\text{emb}}}.$$

Here, $\phi_{\text{GCN}}$ is implemented as a weighted mean aggregation over neighbours


(6) $$e_{i}=\text{ReLU}(\sum_{j\in N(i)}\frac{A_{ij}}{\sum_{l\in N(i)}A_{il}}Px_{j}),$$

Where *P* is a learnable weight matrix, $N(i)=\{j|A_{ij}>0\}$ denotes the neighbourhood of node *i* in *G*, and $x_{j}\in R^{d}$ is the feature vector for node *j* from *X*.

##### 2.3.3.3 Graph attention (GAT) layer

A GAT assignment head (Velickovic *et al.* 2018) projects *E* to cluster logits


(7) $$Z=\phi_{\text{GAT}}(E,A)\in R^{N\times K}.$$

The soft assignment matrix *S* is obtained via row-wise softmax


(8) $$S_{i}{\;}_{k}=\frac{\;\text{exp}(Z_{ik})}{\sum_{l=1}^{K}\;\text{exp}(Z_{il})}.$$

Functionally, the GCN layer encodes the fused graph structure, while the GAT layer implements a differentiable soft partition of nodes into *K* clusters.

#### 2.3.4 Optimisation

To adapt SE minimisation to ST, we augment the loss *H*(*A*; *S*) with a spatial regulariser that encourages neighbouring spatial locations in the spatial graph with adjacency *A*^s^ to have similar assignment distributions. Let *S_i_* denote the *i*-th row of *S*. Inspired by classical total variation (TV) regularisation (Rudin *et al.* 1992), we define a spatial TV penalty as


(9) $$\text{TV}(S)=\frac{{\sum_{i,j}A_{ij}^{s}\left\|S_{i}-S_{j}\right\|}_{1}}{\sum_{i,j}A_{ij}^{s}},$$

Where the sum is over edges in *A*^s^ and ${\left\|\cdot \right\|}_{1}$ denotes the *L*_1_-norm. This term promotes piecewise-smooth domains that respect tissue continuity. The main optimisation objective is therefore


(10) L=H(A;S)+λTV(S),

Where λ controls the strength of spatial regularisation.

We employ a warm-up schedule for λ, allowing the SE term to organise global domains before stronger spatial smoothing is imposed. Training details, including early stopping and post hoc refinement, are provided in Supplementary Sections S4.1–S4.3, available as supplementary data at *Bioinformatics* online. SECTOR also includes an optional cluster-balance penalty $L_{\text{bal}}(S)$ with weight γ, which is activated only when a preliminary probe detects under-use of the specified number of domains; implementation details and practical guidance are provided in Supplementary Section S4.4, available as supplementary data at *Bioinformatics* online. The overall objective combines terms with different gradient scales and is therefore optimised with the Adam optimiser (Kingma and Ba 2015), whose adaptive first- and second-moment updates improve optimisation stability under the TV warm-up schedule and optional balance regularisation. For large-scale datasets such as Visium HD and Xenium, SECTOR uses a scale-aware sparse implementation (Supplementary Section S4.5, available as supplementary data at *Bioinformatics* online), which preserves the same SE, TV and optional balance objectives while avoiding dense N×N distance or adjacency matrices.

### 2.4 Spatiotemporal inference

Upon convergence, SECTOR utilises the learned soft assignments *S* to derive both discrete spatial domains and a continuous pseudotime.

#### 2.4.1 Spatial domain identification

Discrete domains are obtained by assigning each node *i* to the cluster with the maximum posterior probability


(11) $${\hat{y}}_{i}=\underset{k\in \{1,\ldots ,K\}}{\arg\max}S_{ik}.$$

The resulting label vector $\hat{y}$ partitions the tissue into *K* spatially coherent regions.

#### 2.4.2 Pseudotime ordering

To model continuous transitions, we first construct a cluster-level graph with weighted adjacency $A^{c}=S^{T}A^{s}S$. Off-diagonal entries of *A*^c^ represent the total boundary weight between clusters. We then compute the Fiedler vector $\tau \in R^{K}$ (the eigenvector corresponding to the second smallest eigenvalue) of the normalised graph Laplacian of *A*^c^, establishing a spectral ordering of domains. Graph Laplacian eigenvectors, and in particular the Fiedler vector, have previously been used to define one-dimensional trajectories by ordering cells along a cell–cell graph in single-cell datasets (Damo *et al.* 2023, Venkat *et al.* 2024). SECTOR adopts this idea at the level of spatial domains. This cluster-level pseudotime is back-projected to nodes via the soft assignment


(12) $$t=S\tau \in R^{N},$$

and refined via spatial smoothing to reduce local noise. The final pseudotime is oriented using a spatial anchor or root cluster and rescaled to [0,1] (details in Supplementary Section S4.6, available as supplementary data at *Bioinformatics* online).

### 2.5 Benchmark design and evaluation

Spatial clustering (SC) benchmarking was performed on the seven benchmark datasets described in Section 2.1, grouped as sequencing-based ST, imaging-based ST, and modern large-scale high-resolution ST. This grouped presentation enabled concise comparison across distinct data regimes and is consistent with recent studies that report results by technology or dataset scale (Schaub *et al.* 2025, Yuan *et al.* 2024).

We compared SECTOR with three spatiotemporal methods, i.e. CASCAT, stLearn and SpaceFlow, that jointly perform spatial clustering and pseudotime inference (SC+PT) and seven SC-focused methods, i.e. NichePCA, SpaSEG, CellCharter, MENDER, NicheCompass, BANKSY, GraphST. The last five methods were included as strong contemporary baselines highlighted by Dong *et al.* (2025), while NichePCA and SpaSEG were added to further represent recent clustering-focused advances. For the large-scale high-resolution ST group, comparisons were restricted to methods feasible at this scale under the computational resources used in this study. We also report an extended supplementary comparison with earlier but widely used spatial clustering methods on the standard benchmark datasets shared with SDMBench, using the released per-slice metrics from Yuan *et al.* (2024).

In principle, SC methods can be combined post hoc with general single-cell trajectory tools (e.g. Slingshot or Monocle) to construct spatiotemporal analysis pipelines. However, there is currently no standard ST workflow for such combinations, and the design space—with choices regarding clustering method, trajectory tool, graph construction, spatial weighting and root selection—is large. We therefore restricted SC+PT comparisons to methods that natively infer spatially aware pseudotime, while using specialised SC methods as domain-detection baselines.

Following Yuan *et al.* (2024), we evaluated clustering accuracy using normalised mutual information (NMI), homogeneity (HOM) and completeness (COM), and additionally compared runtime and peak memory usage. For the sequencing- and imaging-based ST groups, statistical significance between SECTOR and the SC+PT baselines was assessed using two-sided paired Wilcoxon signed-rank tests on slice-level metrics, with Benjamini–Hochberg correction. Significance testing was not applied to the large-scale high-resolution group because it contains only four Visium HD/Xenium representations. For method parameterisation, we followed Dong *et al.* (2025): dataset-specific recommended settings from the original authors were used when available; otherwise, we searched within the recommended parameter space to identify a strong configuration. When paired histology images were unavailable or image-aware processing was impractical at scale, stLearn was run without its optional morphology-aware workflow, i.e. in its expression-plus-spatial-distance configuration. We did not perform exhaustive dataset-specific tuning for every baseline, so more extensive method-specific tuning could alter performance on individual datasets.

To assess recovery of spatiotemporal organisation, we performed case studies on HER2ST breast cancer and mouse OB. We evaluated clustering accuracy and pseudotime smoothness, the latter quantified by spatial semivariance (Pham *et al.* 2023), and supported the results with downstream computational analyses, including pseudotime–gene correlation and Gene Ontology (GO) enrichment for HER2ST, and supertype-resolved UMAP and marker-gene profiling for mouse OB. Further benchmarking and case study details are provided in Supplementary Section S5, available as supplementary data at *Bioinformatics* online.

## 3 Results

### 3.1 Overview

SECTOR is a unified deep graph learning framework for inferring spatial domains and pseudotime from a single ST section (Fig. 1). It constructs a fused spatial–expression graph from physical neighbourhoods and transcriptomic similarity, then learns soft domain assignments by minimising a differentiable SE objective on a two-layer graph-encoding tree, represented as root–clusters–spatial locations. Spatial TV regularisation promotes locally coherent domains, and an optional balance penalty is used when needed to reduce severe under-use of the specified number of domains. Discrete domains are obtained from the learned assignments, whereas pseudotime is inferred from the induced cluster-level graph and propagated to individual locations. This pseudotime represents a latent within-section progression axis rather than directly observed chronological time. For large-scale datasets such as Visium HD and Xenium, SECTOR uses a sparse implementation that preserves the same optimisation objective while improving scalability. Together, these design choices make SECTOR lightweight and applicable across diverse ST platforms.

### 3.2 SECTOR achieves consistently strong spatial clustering performance across diverse ST technologies

**Figure 2:**
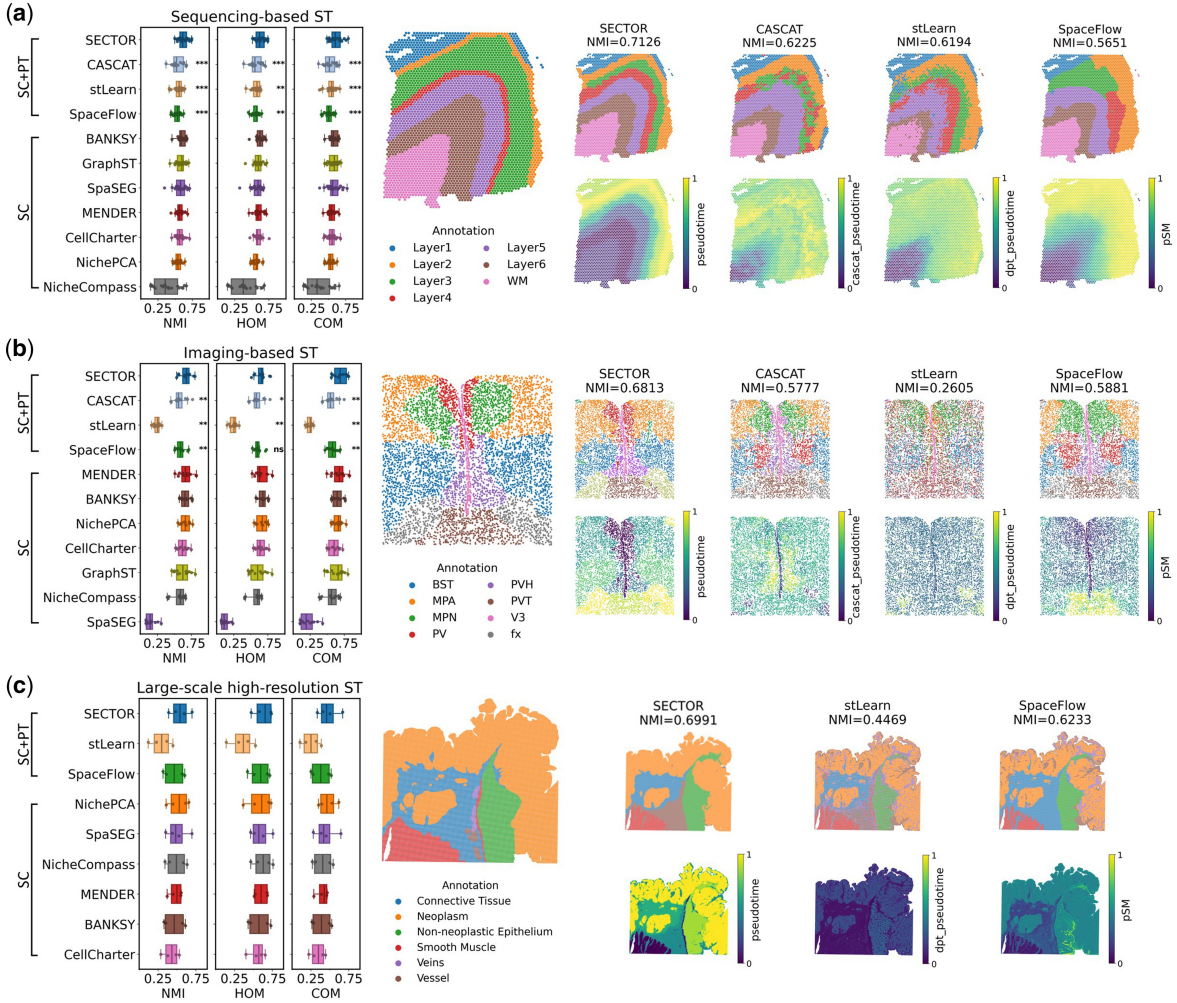
Benchmarking spatial clustering performance across diverse ST technologies. SECTOR was compared with spatiotemporal methods that jointly perform spatial clustering and pseudotime inference (SC+PT) and spatial clustering-only methods (SC) using normalised mutual information (NMI), homogeneity (HOM), and completeness (COM). In the boxplots, SC+PT methods are shown in a fixed order for consistent comparison, whereas SC methods are ordered by descending median NMI within each dataset group. (a) Sequencing-based ST benchmarks, including 10x Visium DLPFC (Maynard *et al*. 2021) and Stereo-seq mouse embryos (Chen *et al*. 2022); representative spatial maps show annotation, inferred domains and pseudotime on DLPFC slice 151673. (b) Imaging-based ST benchmarks, including MERFISH mouse hypothalamus (Moffitt *et al*. 2018), STARmap cortex (Wang *et al*. 2018) and BaristaSeq primary cortex (Long *et al*. 2023); representative maps show MERFISH slice Bregma = −0.24. Significance markers in (a, b) compare SECTOR with SC+PT baselines using two-sided paired Wilcoxon signed-rank tests on slice-level metrics with Benjamini–Hochberg correction: ^∗^*P *< 0.05, ^∗∗^*P *< 0.01, ^∗∗∗^*P *< 0.001, and ns, non-significant. (c) Large-scale high-resolution ST benchmarks, including Visium HD CRC (Oliveira *et al*. 2025) and Xenium IDC (Bhuva *et al*. 2024); representative maps show the Visium HD CRC 16-μm binned output. Significance testing was not applied in (c) because this group contains only four Visium HD/Xenium representations.

Figure 2 summarises the benchmark comparison of SECTOR with SC+PT and SC baselines across the three dataset groups defined in Section 2.1. Statistical significance against the SC+PT baselines is annotated in Fig. 2a, b. Representative spatial domain and pseudotime outputs are shown in Fig. 2 and Figs. S1–S3, available as supplementary data at *Bioinformatics* online, and extended comparisons including earlier widely used baselines from Yuan *et al.* (2024) are provided in Fig. S4, available as supplementary data at *Bioinformatics* online.

For the sequencing-based ST group, including 10x Visium DLPFC and Stereo-seq mouse embryo, SECTOR ranked first across NMI, HOM and COM (Fig. 2a) and was significantly better than all three SC+PT baselines. On the representative DLPFC slice 151,673 SECTOR attained the highest NMI (0.7126), outperforming CASCAT (0.6225), stLearn (0.6194) and SpaceFlow (0.5651), while also remaining ahead of recent SC baselines shown in Fig. S1a, available as supplementary data at *Bioinformatics* online. Qualitatively, SECTOR reconstructed the cortical laminar organisation, delineating white matter and cortical layers with smooth boundaries. Beyond discrete domains, SECTOR recovered an anatomically plausible laminar pseudotime gradient aligned with cortical depth: the inferred pseudotime formed a relatively smooth, approximately monotonic gradient from the deep cortical layers toward the superficial layers, with L6 occupying the start of the gradient. On Stereo-seq, differences between methods were visually smaller (Figs. S1b and S4b, available as supplementary data at *Bioinformatics* online) because many methods recovered coarse embryonic compartments similarly, whereas finer or less abundant structures remained challenging owing to high sparsity (Yuan *et al.* 2024) and complex embryonic annotations.

The imaging-based ST benchmarks, including MERFISH hypothalamus, STARmap cortex and BaristaSeq primary cortex, showed a similar trend (Fig. 2b). Compared with the SC+PT baselines, SECTOR was significantly better for NMI and COM across the group, and for HOM except against SpaceFlow. We interpret this to mean that SECTOR’s advantage over SpaceFlow in imaging-based ST is more consistent for overall clustering agreement and completeness than for cluster homogeneity. On the representative MERFISH slice (Bregma = −0.24), SECTOR achieved the highest NMI (0.6813), exceeding CASCAT (0.5777), stLearn (0.2605) and SpaceFlow (0.5881), and remaining ahead of strong SC baselines (Fig. S2a, available as supplementary data at *Bioinformatics* online). Qualitatively, SECTOR recovered the major hypothalamic compartments with cleaner transitions near the midline and produced a coherent radial pseudotime gradient: low pseudotime values were concentrated along the central V3–PVH ventricular axis and increased smoothly toward the lateral and ventral periphery. By contrast, the SC+PT baselines produced less spatially coherent or less anatomically aligned gradients. Representative STARmap and BaristaSeq slices showed a similar pattern, with SECTOR producing cleaner spatial domains and more coherent pseudotime fields than the SC+PT baselines (Fig. S2b, c, available as supplementary data at *Bioinformatics* online).

For the modern large-scale high-resolution ST group, SECTOR exhibited the strongest overall performance among methods feasible at Visium HD/Xenium scale (Fig. 2c). CASCAT and GraphST were not included because CASCAT’s conditional-mutual-information-based causal pruning and GraphST’s whole-graph contrastive GNN training were impractical for these datasets. On the representative Visium HD CRC 16-μm binned output, SECTOR achieved the highest NMI (0.6991), exceeding SpaceFlow (0.6233), stLearn (0.4469) and the SC baselines (Fig. 2c and Fig. S3a, available as supplementary data at *Bioinformatics* online). SECTOR produced cleaner and more contiguous domains, particularly near interfaces between neoplasm, connective tissue and non-neoplastic epithelium, and inferred a broad pseudotime gradient aligned with the overall tissue architecture. On the representative Xenium IDC cell-level section (Fig. S3b, available as supplementary data at *Bioinformatics* online), which is more challenging due to finer spatial granularity and stronger local heterogeneity, SECTOR again achieved stronger clustering metrics and produced domains that better matched the annotated invasive tumour, DCIS, normal duct and stromal regions. Its pseudotime field further captured a clearer within-section progression-associated gradient spanning DCIS/normal duct-rich regions and the invasive tumour compartment than those inferred by stLearn and SpaceFlow.

Overall, SECTOR consistently outperformed the SC+PT baselines, while remaining competitive with, or superior to, specialised SC methods across the three benchmark groups.

### 3.3 SECTOR improves spatiotemporal inference on HER2ST and mouse OB datasets

**Figure 3:**
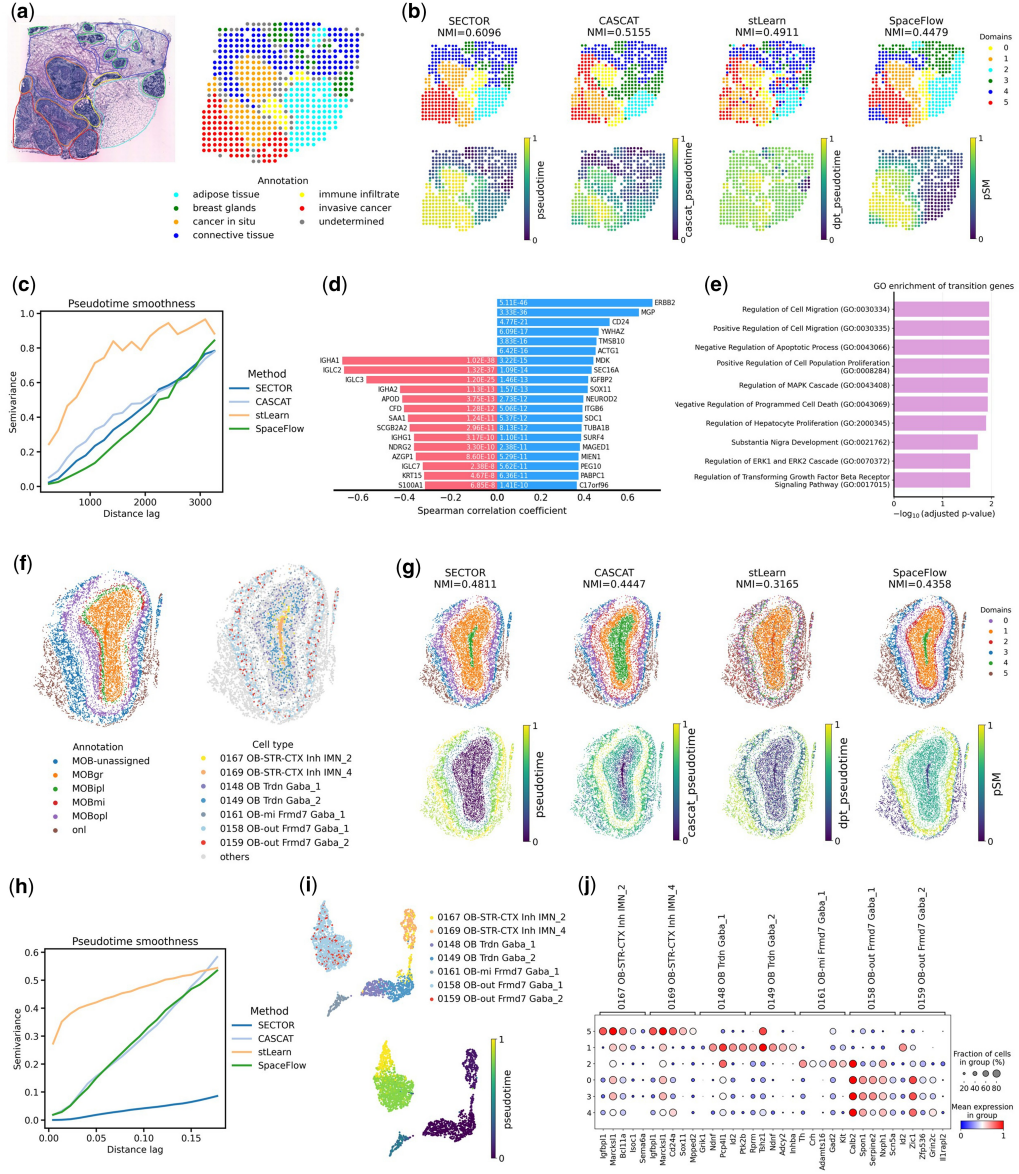
Recovery of spatiotemporal organisation in human breast cancer progression and mouse olfactory bulb (OB) development. (a–e) The 10x Visium HER2ST breast cancer section (Andersson *et al*. 2021): (a) H&E image and pathologist annotations. (b) Spatial domains and pseudotime inferred by SECTOR and spatiotemporal baselines (CASCAT, stLearn and SpaceFlow). (c) Semivariance of pseudotime as a function of spatial distance lag (lower values indicate smoother pseudotime). (d) Top transition genes positively (blue) and negatively (red) correlated with SECTOR pseudotime. (e) Gene Ontology (GO) enrichment of SECTOR transition genes. (f–j) The MERFISH mouse OB section (Zhang *et al*. 2023): (f) Reference annotations and developmental cell types. (g) Spatial domains and pseudotime inferred by SECTOR and spatiotemporal baselines. (h) Semivariance of pseudotime across distance lags. (i) UMAP embeddings coloured by cell type (top) and SECTOR pseudotime (bottom). (j) Dot plot of marker gene expression across SECTOR-identified domains, illustrating the transition from immature (domains 1 and 5) to mature (0, 3 and 4) OB neuronal states.

We first investigated recovery of continuous cancer progression using the 10x Visium HER2ST slice, which contains invasive cancer, cancer in situ, immune infiltrates, breast glands, connective tissue and adipose tissue (Fig. 3a). SECTOR achieved the highest concordance with pathologist annotations (NMI = 0.6096), outperforming CASCAT (0.5155), stLearn (0.4911) and SpaceFlow (0.4479) (Fig. 3b, top). Under the benchmark settings used in this study, SECTOR more clearly delineated the boundary between invasive and in situ regions, whereas this distinction was less evident in the outputs of CASCAT, stLearn, and SpaceFlow. SECTOR also inferred a smooth pseudotime field spanning the tissue architecture (Fig. 3b, bottom). Spatial semivariance analysis showed that CASCAT and SpaceFlow produced curves broadly similar to SECTOR, whereas stLearn exhibited substantially higher semivariance (Fig. 3c). Importantly, semivariance captures only spatial smoothness, not the full biological fidelity of the inferred pseudotime. In HER2ST, the dominant pathological gradient is broad and spatially contiguous, so multiple methods can generate similarly smooth fields even when they differ in clustering accuracy and biological relevance. SECTOR therefore stands out not because it is uniquely smooth in this case, but because it combines low semivariance with the highest agreement to pathologist annotations.

To assess the biological relevance of the inferred HER2ST pseudotime, we identified “transition genes” significantly correlated with SECTOR’s pseudotime (adjusted *P *< 0.05 and Spearman correlation >0.3 or <−0.3). ERBB2 displayed the strongest positive correlation (Fig. 3d); beyond this primary driver (Ng *et al.* 2015), SECTOR identified key malignancy-associated markers including MGP, CD24 and YWHAZ, which were ranked lower by competing methods (Fig. S5, available as supplementary data at *Bioinformatics* online). Conversely, immunoglobulin genes (IGHA1 and IGLC2) showed strong negative correlations, consistent with a trajectory running from immune-infiltrated connective tissue toward the tumour core. GO enrichment of SECTOR-derived transition genes highlighted that the inferred pseudotime captured molecular programmes related to tumour aggressiveness, including “regulation of cell migration” and “MAPK cascade” (Fig. 3e). In contrast, transition genes identified by baselines were enriched for more generic or less tissue-relevant terms (Fig. S5, available as supplementary data at *Bioinformatics* online). These results support the pathological relevance of the pseudotime axis inferred by SECTOR.

Next, we assessed developmental resolution using the MERFISH mouse OB section, which exhibits a nonlinear radial maturation pattern and stronger local heterogeneity. SECTOR again achieved the highest clustering accuracy (NMI = 0.4811), outperforming CASCAT (0.4447), SpaceFlow (0.4358) and stLearn (0.3165) (Fig. 3g). Qualitatively, SECTOR’s domains aligned with the spatial distribution of immature neuron types (0167, 0169) in the tissue core (Fig. 3f). Leveraging this structure, SECTOR inferred a smooth, radial pseudotime that progressed from these central immature populations toward the mature periphery (Fig. 3g, bottom). By contrast, the baseline methods produced noisier, less radial or less anatomically aligned pseudotime patterns. In mouse OB, semivariance curves showed a much larger separation between SECTOR and the baselines (Fig. 3h) than in HER2ST (Fig. 3c). This suggests that smoothness is more discriminative when the expected progression pattern is confined to a narrower region of the tissue and radially organised. Methods that aligned less well with the immature-to-mature neuronal axis produced more irregular local fluctuations and therefore higher semivariance. This stronger contrast likely reflects both dataset-specific biological organisation and the finer spatial granularity of MERFISH, although these effects cannot be fully disentangled in this comparison.

Finally, uniform manifold approximation and projection (UMAP) analysis shows that SECTOR recovered a continuous manifold (Fig. 3i, top) in which the pseudotime gradient closely aligned with the known lineage from immature inhibitory neurons (0167 and 0169) to mature GABAergic interneurons (0158 and 0159) (Fig. 3i, bottom). This differentiation axis was further supported by the expression profiles of SECTOR’s spatial domains (Fig. 3j): early-stage clusters (1 and 5) were enriched for immaturity-associated markers (Igfbpl1 and Bcl11a), whereas late-stage clusters (0, 3 and 4) were enriched for maturation-associated markers (Calb2 and Kit). Baseline methods resolved these states less clearly, either mixing distinct populations or showing greater marker overlap between domains (Fig. S6, available as supplementary data at *Bioinformatics* online).

### 3.4 Parameter sensitivity and computational efficiency

**Figure 4:**
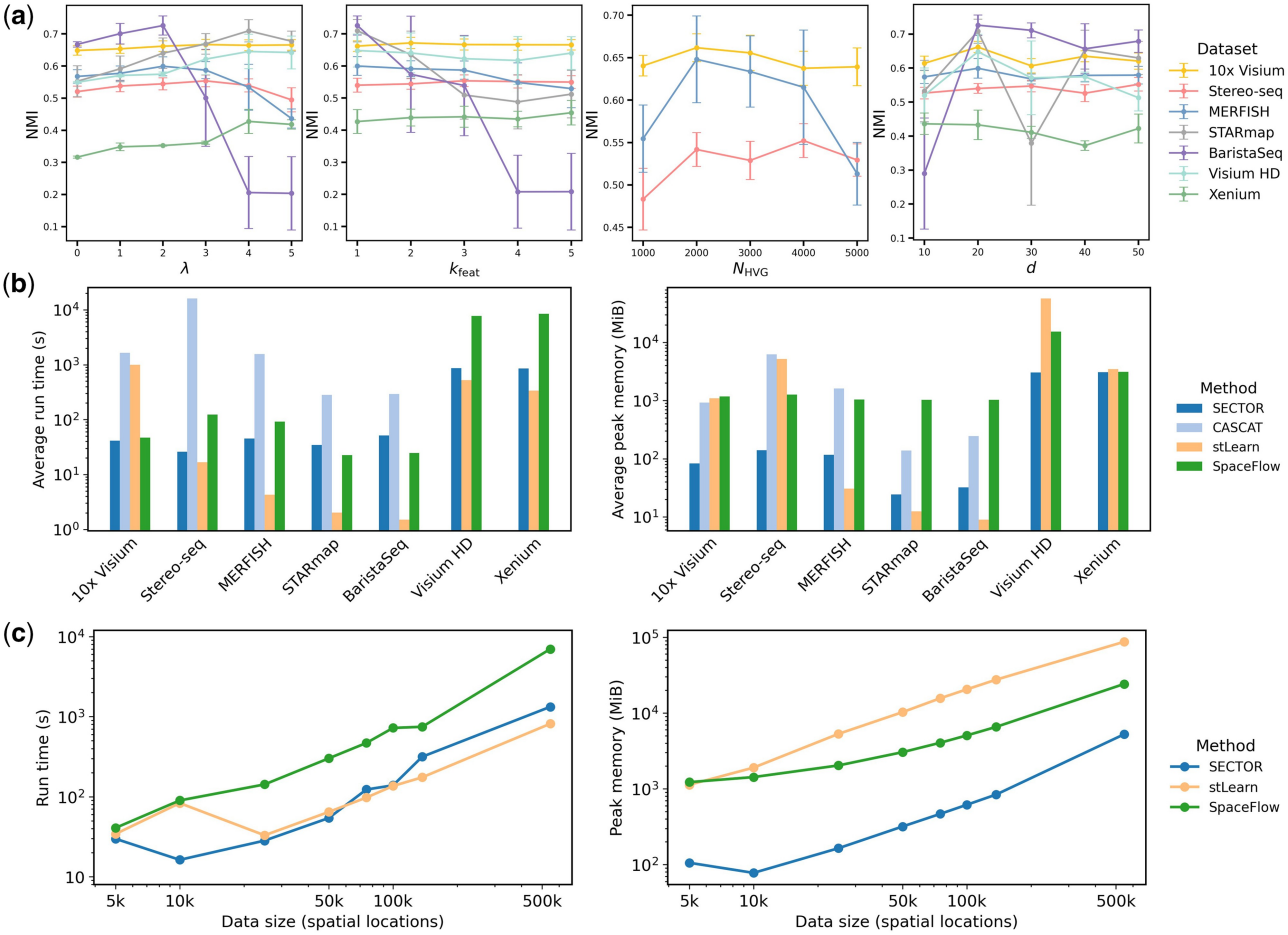
Parameter sensitivity and computational efficiency. (a) Sensitivity analysis of SECTOR’s spatial clustering accuracy (NMI) across the benchmark datasets. Hyperparameters evaluated include the spatial regularisation weight (*λ*), the number of feature-graph neighbours ($k_{\text{feat}}$), the number of HVGs ($N_{\text{HVG}}$), and the PCA dimension (*d*). (b) Comparison of runtime (left) and peak memory (right) between SECTOR and spatiotemporal baselines (CASCAT, stLearn and SpaceFlow) on the same datasets. (c) Controlled within-sample scaling analysis on the Visium HD CRC dataset: runtime (left) and peak memory (right) of SECTOR, stLearn and SpaceFlow as the data size increases from 5,000 to 545,909 spatial locations.

To assess SECTOR’s robustness, we analysed four key parameters across the seven benchmark datasets: the spatial TV coefficient λ, feature-graph neighbour number $k_{\text{feat}}$, number of HVGs $N_{\text{HVG}}$, and PCA dimension *d* (Fig. 4a). SECTOR was stable across moderate parameter ranges, supporting the default settings (Supplementary Section S4.1, available as supplementary data at *Bioinformatics* online) as practical starting points. λ was the most dataset-sensitive parameter: moderate smoothing generally preserved or improved performance, whereas excessive smoothing reduced NMI on BaristaSeq, likely because its small targeted gene panel and narrow adjacent laminar domains make it vulnerable to boundary blurring between cortical layers and white matter. Conversely, STARmap, Visium HD and Xenium benefited from stronger spatial regularisation, suggesting that additional smoothing can help suppress local noise or fragmentation in sparse, high-resolution or heterogeneous datasets. Low feature-graph connectivity was generally sufficient, with $k_{\text{feat}}=1$ performing well across most datasets, while larger values reduced accuracy on STARmap and BaristaSeq, suggesting that overly dense expression-neighbour graphs can introduce noisy or less biologically relevant connections. $N_{\text{HVG}}$ also affected performance for large-panel or whole-transcriptome datasets, with 2,000–4,000 HVGs providing a robust range; targeted-panel datasets typically retained most or all informative genes and were therefore less dependent on this parameter. The PCA dimension *d* had a weaker overall effect, with *d *= 20 providing a stable default across datasets.

Additional sensitivity analyses supported SECTOR’s default design. Replacing HVGs with Moran’s I-ranked spatially variable genes (SVGs) (Túrós *et al.* 2024) did not improve clustering accuracy (Supplementary Section S6, available as supplementary data at *Bioinformatics* online). This likely reflects that SECTOR already encodes spatial information through the fused graph and TV regularisation, whereas SVG-based feature selection may discard domain-discriminative transcriptomic signals. Gene-panel downsampling on the Visium HD CRC 16-μm binned output indicated that SECTOR remained robust with 3000 or 1500 genes, with clear degradation mainly at 500 and 250 genes (Supplementary Section S7, available as supplementary data at *Bioinformatics* online), suggesting that panels in the low thousands can still support reliable spatial clustering when informative genes are retained. Ablation analysis further indicated that optional components, including the cluster-balance penalty, post hoc refinement and early stopping, can support stable domain recovery in selected settings (Supplementary Section S8, available as supplementary data at *Bioinformatics* online).

We next compared computational efficiency across datasets by recording average runtime and peak memory usage of the spatiotemporal methods (Fig. 4b). On the standard benchmark datasets, SECTOR was substantially faster than CASCAT and generally faster than SpaceFlow, while maintaining a small memory footprint. stLearn often had the shortest runtime on datasets without paired histology images, where it was run in its expression-plus-spatial-distance configuration. On the large-scale Visium HD and Xenium datasets, SECTOR remained practical, with lower or comparable memory usage relative to stLearn and SpaceFlow and competitive runtime. CASCAT was not evaluated at this scale because its causal-pruning step was impractical for these datasets.

To reduce confounding from platform and tissue context, we further performed a controlled within-sample scaling analysis using the Visium HD CRC dataset (Fig. 4c). Spatially contiguous central subsets of the 16-µm binned output were generated (Supplementary Section S5.2, available as supplementary data at *Bioinformatics* online), increasing the number of spatial locations from 5000 to 137 048; the 8-µm binned output was included to extend the analysis to 545 909 bins. Across this range, SECTOR maintained a more favourable memory profile than the baselines and remained faster than SpaceFlow. Its runtime was comparable to stLearn, although stLearn was faster at larger data sizes under the expression-plus-spatial-distance configuration. Overall, these results support SECTOR’s practical scalability across the tested range.

## 4 Discussion

This study presents SECTOR, a unified deep graph learning framework for spatial domain detection and pseudotime inference. Classical SE (Li and Pan 2016) was originally defined on discrete graph partitions or partitioning trees and minimised through combinatorial procedures. More recently, stTrace (Song *et al.* 2025) applied classical SE to ST trajectory analysis, while assuming a precomputed developmental level for each spot or cell. DeSE (Zhang *et al.* 2025) introduced a differentiable SE objective for generic graph clustering based on binary graph structures, edge-based reconstruction, and stacked GNN assignment layers. SECTOR differs from these approaches by reformulating SE as a lightweight, ST-specific differentiable objective on a fused weighted spatial–expression graph, modelled through a two-layer graph-encoding tree and regularised by spatial TV. These design choices enabled strong spatial domain recovery and biologically interpretable pseudotime across seven benchmark datasets and two case studies.

SECTOR nonetheless has practical limitations. Its performance depends on dataset-specific parameters (Fig. 4a), including the spatial regularisation strength, feature-graph connectivity, HVG number and PCA dimension, which can interact with optimisation-related parameters such as learning rate, early stopping criteria, and cluster-balance weight. The default settings provide practical starting points, but modest adjustment may be beneficial when outputs appear oversmoothed, fragmented, unstable or when clusters are under-used. SECTOR also depends on preprocessing and fused graph construction, especially in the large-scale mode used for Visium HD and Xenium, where sparse nearest-neighbour graphs improve scalability but make performance more dependent on initial graph quality. Moreover, low-coverage or highly heterogeneous tissues may require careful spatial regularisation, as excessive smoothing can blur narrow adjacent regions whereas insufficient smoothing may fragment noisy domains. Finally, multi-million-scale data, such as the Visium HD CRC 2-μm binned output with over eight million bins (Supplementary Section S1.1.6, available as supplementary data at *Bioinformatics* online), and integrated multi-section or multi-time-point analyses remain beyond the current implementation.

SECTOR currently infers pseudotime from a single slice and therefore recovers a latent progression axis rather than directly observed chronological time. Genuine temporal dynamics are more directly addressed with aligned cross-time-point or longitudinal multi-section ST data. Extending SECTOR to such settings will require more than scaling with node number alone: sections should be reliably integrated and aligned in a common coordinate framework while mitigating pose variation, geometric deformation and slicing bias (Dong *et al.* 2025, Heitz *et al.* 2025), thereby enabling more faithful modelling of development, ageing and other temporally evolving processes. A practical extension could construct block-structured sparse graphs with within-section edges and cross-section correspondence edges, combined with memory-efficient sparse processing and explicit temporal or branch-aware graph modelling.

Additional extensions could further broaden SECTOR’s applicability. Although SECTOR was designed to operate without auxiliary image data, its fused-graph architecture could incorporate histology-derived morphology features, for example by adding a morphology graph from image embeddings or augmenting the transcriptomic feature representation before graph construction. Related methods such as stLearn (Pham *et al.* 2023) and Chrysalis (Túrós *et al.* 2024) suggest that morphology can complement transcriptomic information in tissues with strong histological structure. SECTOR may also benefit from emerging spatial foundation models such as Novae (Blampey *et al.* 2025), whose transferable embeddings across tissues, gene panels and technologies could replace or augment SECTOR’s current PCA-based representation. In multi-slice settings, such embeddings may provide informative inputs for joint clustering and trajectory inference while preserving SECTOR’s core SE-based formulation.

## Supplementary Material

btag367_Supplementary_Data

## Data Availability

SECTOR is available on GitHub at https://github.com/lhbcb/SECTOR. The version used for the analyses reported here has been archived on Figshare at https://doi.org/10.6084/m9.figshare.32029830, together with the benchmark reproduction code, case-study notebooks, tutorial notebooks, and processed datasets generated in this study. Details of raw and curated dataset sources are provided in Supplementary Section S1, available as supplementary data at *Bioinformatics* online.
