# Supplementary material for: SECTOR: structural entropy-based learning of spatiotemporal organisation in spatial transcriptomics
Source: Bioinformatics. 2026 Jun 15;42(6):btag367. doi: 10.1093/bioinformatics/btag367 (PMC13303290; doi:10.1093/bioinformatics/btag367)
Supplement: btag367_Supplementary_Data [file btag367_supplementary_data.pdf]

Supplementary Information for:

SECTOR: structural entropy-based learning of spatiotemporal organisation in spatial transcriptomics

Li Huang[1,†], Jingyun Zhang[2,†], Weikang Gong[1], Guangjie Zeng[3], Hao Peng[2,*] and Dongsheng Chen[1,*]

[1]State Key Laboratory of Common Mechanism Research for Major Diseases, Suzhou Institute of Systems Medicine, Chinese Academy of Medical Sciences and Peking Union Medical College, Suzhou 215123, China

[2]School of Cyber Science and Technology, Beihang University, Beijing 100191, China

[3]School of Computer Science and Engineering, Beihang University, Beijing 100191, China

[†]These authors contributed equally to this work.

*Corresponding authors: cds@ism.pumc.edu.cn; penghao@buaa.edu.cn

Table of Contents

Figure S1 Visual benchmarking on representative sequencing-based ST slices 2

Figure S2 Visual benchmarking on representative imaging-based ST slices 3

Figure S3 Visual benchmarking on representative large-scale high-resolution ST datasets........ 4

Figure S4 Extended benchmarking on standard ST datasets shared with SDMBench 5

Figure S5 HER2ST transition-gene and GO enrichment analyses for spatiotemporal baseline methods........ 6

Figure S6 Mouse OB pseudotime and marker-gene analyses for spatiotemporal baseline methods 7

Section S1 Details of datasets and preprocessing procedures........ 8

Section S2 Details of spatial and feature graph construction........ 11

Section S3 Derivation of two-layer differentiable SE........ 12

Section S4 Details of SECTOR implementation and parametrisation 13

Section S5 Details of benchmarking and case studies 17

Section S6 Comparison of HVG- and SVG-based feature graph construction 19

Section S7 Sensitivity analysis of gene panel size within the Visium HD CRC 16-μm binned output 21

Section S8 Ablation analysis of cluster-balance regularisation, early stopping and post hoc refinement........ 22

References........ 23

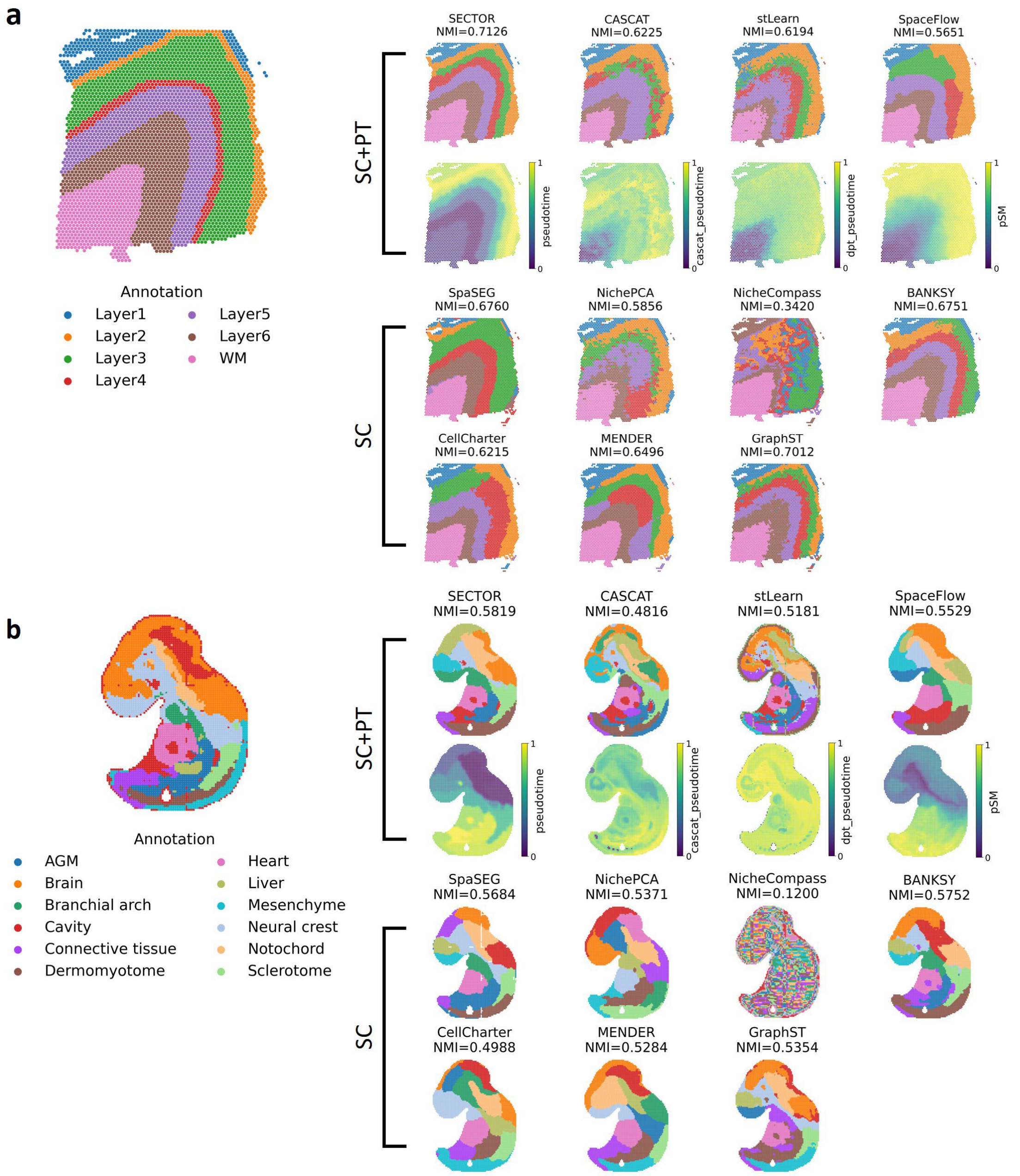

Figure S1. Visual benchmarking on representative sequencing-based ST slices. (**a**) 10x Visium human DLPFC slice 151673. (**b**) Stereo-seq mouse embryo slice E9.5_E1S1.MOSTA. Reference annotations are shown on the left. Predicted spatial domains are shown for SECTOR, spatiotemporal baselines that jointly perform spatial clustering and pseudotime inference (SC+PT: CASCAT, stLearn and SpaceFlow), and spatial clustering-only baselines (SC: SpaSEG, NichePCA, NicheCompass, BANKSY, CellCharter, MENDER and GraphST). Pseudotime fields are shown for SECTOR and the SC+PT baselines. Normalised mutual information (NMI) values are reported above each predicted domain map.

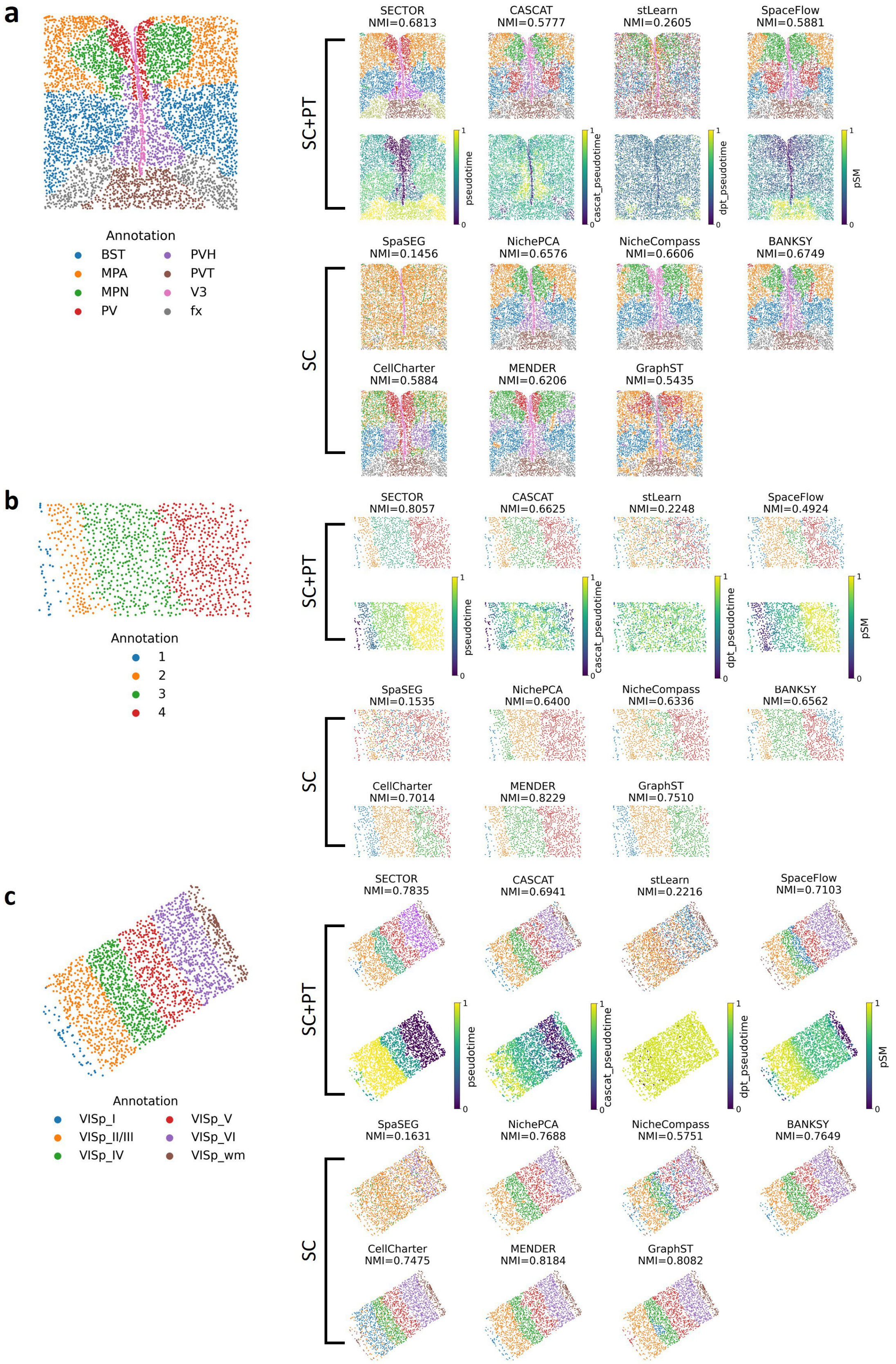

Figure S2. Visual benchmarking on representative imaging-based ST slices. (**a**) MERFISH mouse hypothalamus slice Bregma = −0.24. (**b**) STARmap mouse cortex slice 20180424_BZ14_control. (**c**) BaristaSeq mouse primary visual cortex slice 2. Reference annotations are shown on the left. Predicted spatial domains are shown for SECTOR, spatiotemporal baselines that jointly perform spatial clustering and pseudotime inference (SC+PT: CASCAT, stLearn and SpaceFlow), and spatial clustering-only baselines (SC:

SpaSEG, NichePCA, NicheCompass, BANKSY, CellCharter, MENDER and GraphST). Pseudotime fields are shown for SECTOR and the SC+PT baselines. Normalised mutual information (NMI) values are reported above each predicted domain map.

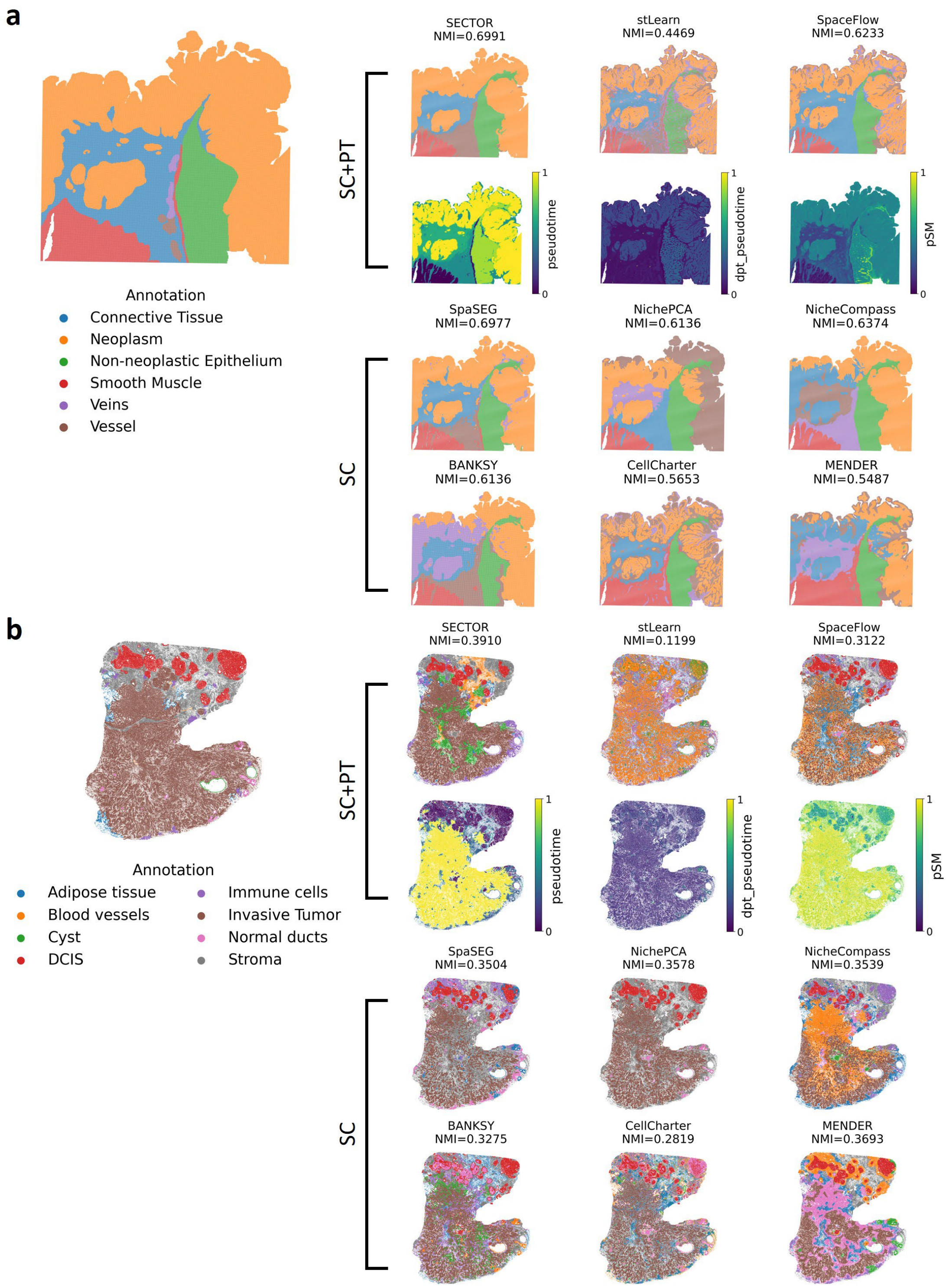

Figure S3. Visual benchmarking on representative large-scale high-resolution ST datasets. (**a**) Visium HD CRC 16-µm binned output. (**b**) Xenium IDC cell-level representation. Reference annotations are shown on the left. Predicted spatial domains are shown for SECTOR, feasible spatiotemporal baselines that jointly perform spatial clustering and pseudotime inference (SC+PT: stLearn and SpaceFlow), and spatial clustering-only baselines (SC: SpaSEG, NichePCA, NicheCompass, BANKSY, CellCharter and MENDER). Pseudotime fields are shown for SECTOR and the SC+PT baselines. Normalised mutual information (NMI) values are reported above each predicted domain map.

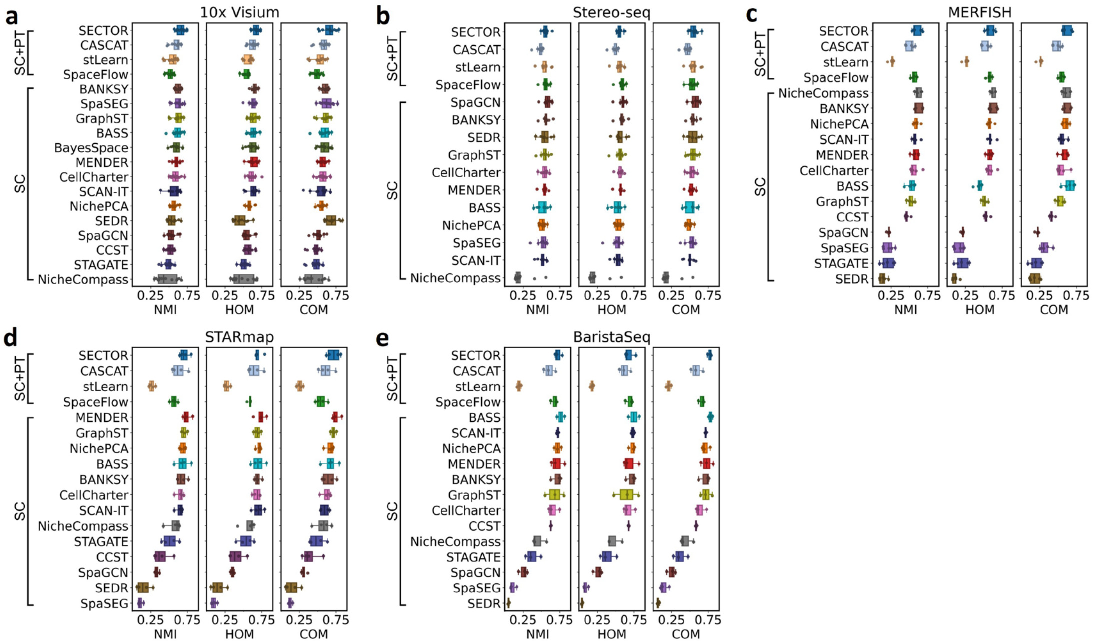

Figure S4. Extended benchmarking on standard ST datasets shared with SDMBench. Boxplots show slice-level normalised mutual information (NMI), homogeneity (HOM) and completeness (COM) for SECTOR and the primary baselines evaluated in this study, including SC+PT methods (CASCAT, stLearn and SpaceFlow) and SC methods (CellCharter, MENDER, NicheCompass, BANKSY, GraphST, NichePCA and SpaSEG). Released per-slice metrics from Yuan *et al.* (2024) are also included for earlier spatial clustering baselines: SpaGCN, SCAN-IT, STAGATE, CCST, SEDR, BayesSpace and BASS. Panels show boxplots for (**a**) 10x Visium human DLPFC, (**b**) Stereo-seq mouse embryo, (**c**) MERFISH mouse hypothalamus, (**d**) STARmap mouse cortex and (**e**) BaristaSeq mouse primary visual cortex. In each panel, SC+PT methods are shown in a fixed order (SECTOR, CASCAT, stLearn and SpaceFlow) for consistent comparison, whereas SC methods are ordered by descending median NMI.

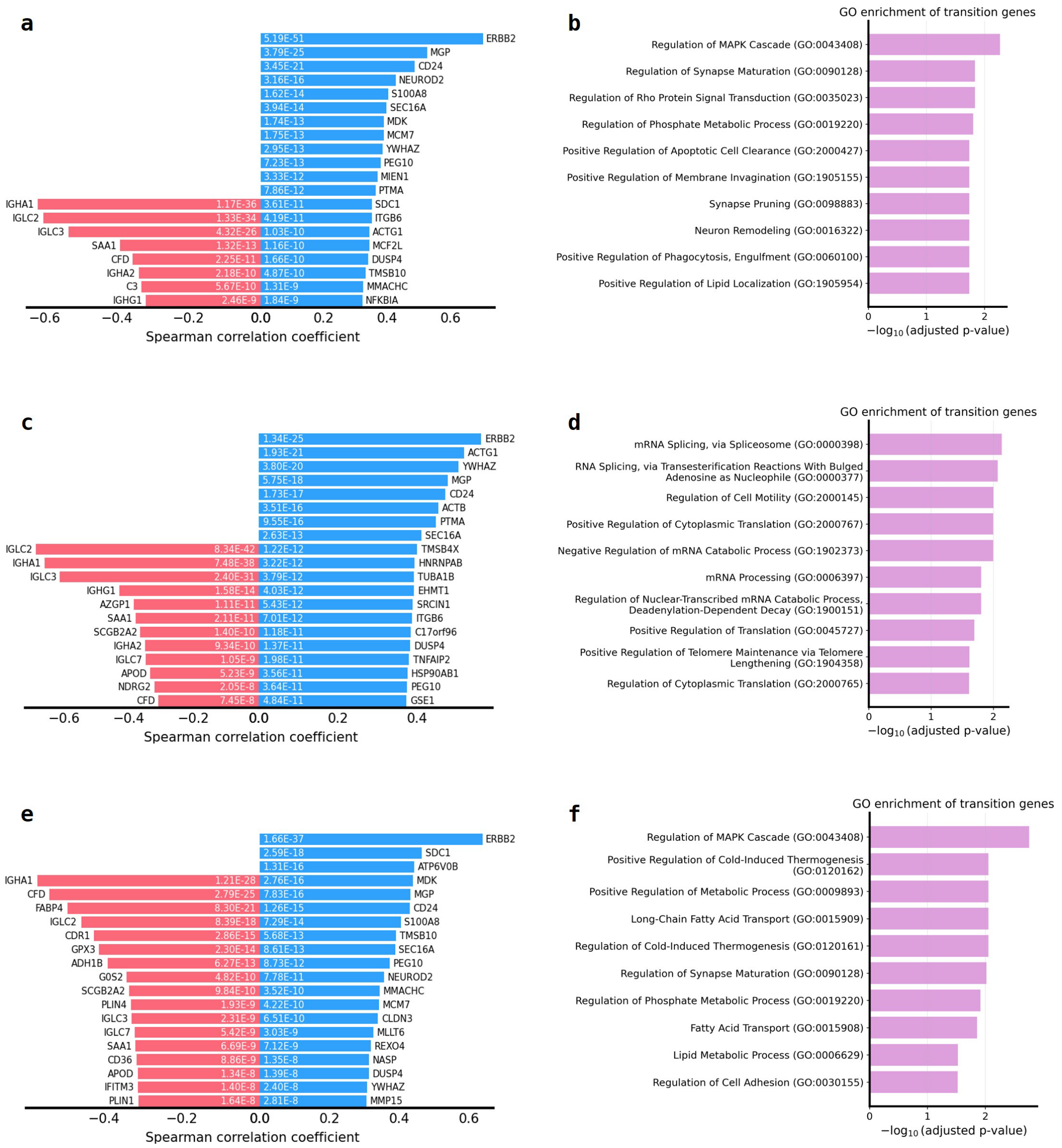

Figure S5. HER2ST transition-gene and Gene Ontology (GO) enrichment analyses for spatiotemporal baseline methods. (**a,b**) CASCAT, (**c,d**) stLearn and (**e,f**) SpaceFlow. (**a,c,e**) Top transition genes ranked by Spearman correlation between gene expression and each method's inferred pseudotime; blue and red bars indicate positive and negative correlations, respectively, with adjusted *p* values shown on the bars. (**b,d,f**) GO Biological Process enrichment of significant transition genes identified by each baseline method. Compared with SECTOR (Fig. 3d,e), baseline-derived transition genes were enriched for more generic or less tumour-progression-related processes.

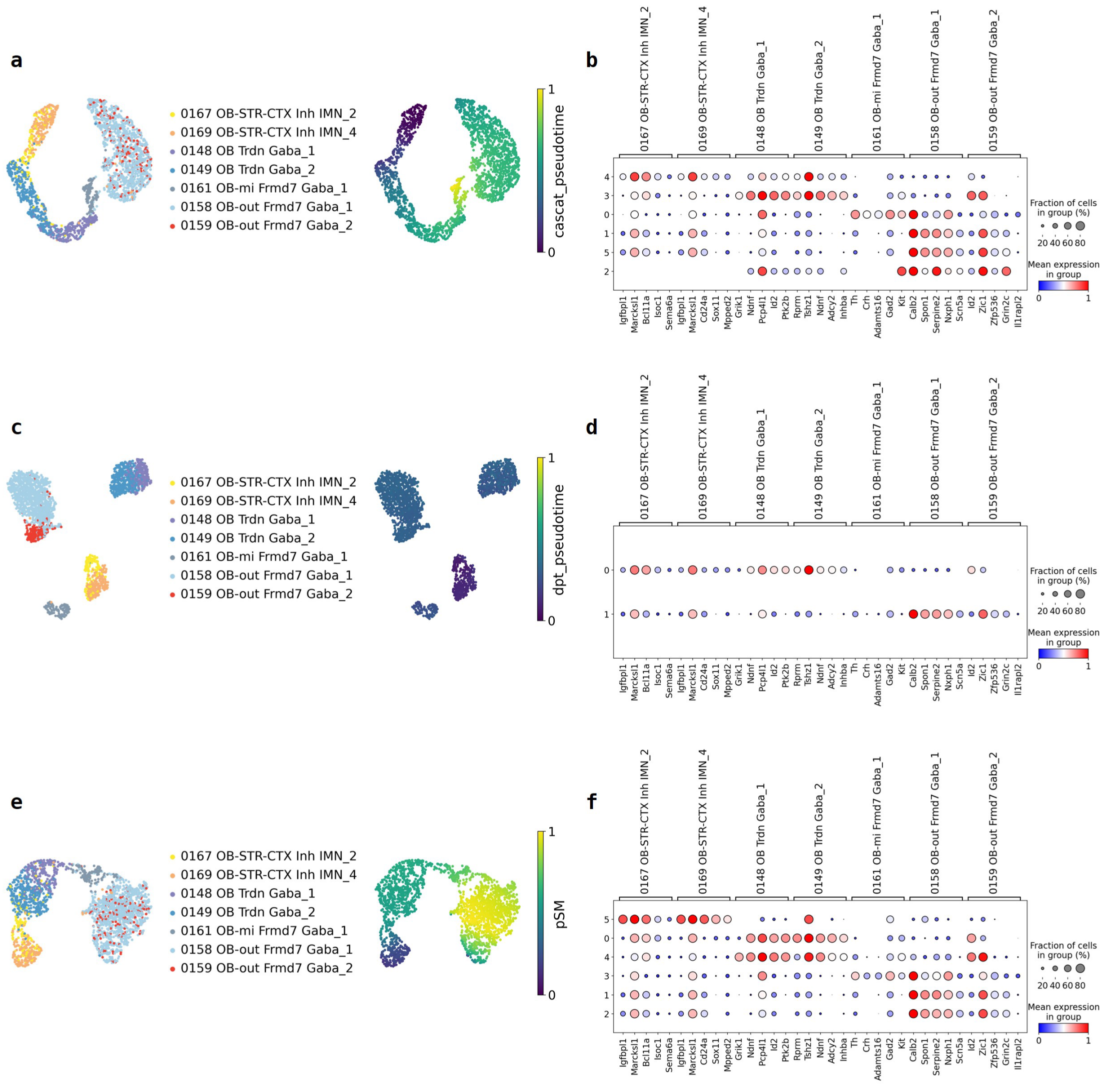

Figure S6. Mouse olfactory bulb (OB) pseudotime and marker-gene analyses for spatiotemporal baseline methods. (**a,b**) CASCAT, (**c,d**) stLearn and (**e,f**) SpaceFlow. (**a,c,e**) UMAP embeddings of the mouse olfactory bulb (OB) dataset coloured by OB inhibitory-neuron supertype annotation and by each method's inferred pseudotime. (**b,d,f**) Dot plots showing expression of representative developmental marker genes across the spatial domains predicted by each baseline method; dot size indicates the fraction of cells expressing each gene and colour indicates scaled mean expression. Compared with SECTOR (Fig. 3i,j), the baseline methods showed less clear resolution of the OB neuronal maturation axis, with greater mixing of immature and mature populations or weaker separation of marker-gene profiles across domains.

S1 Details of datasets and preprocessing procedures

In this study, we used nine publicly available spatial transcriptomics (ST) datasets spanning sequencing-based and imaging-based platforms. Seven datasets were used for spatial clustering benchmarking: 10x Visium DLPFC, Stereo-seq mouse embryo, MERFISH hypothalamus, STARmap cortex, BaristaSeq primary cortex, Visium HD colorectal cancer (CRC) and Xenium infiltrating ductal carcinoma (IDC). These datasets were selected to cover established benchmark datasets as well as modern large-scale high-resolution ST platforms. Two additional datasets, HER2ST breast cancer and mouse olfactory bulb (OB) MERFISH, were used as case studies for within-section pseudotime inference and downstream biological interpretation.

S1.1 Datasets for benchmarking spatial domain detection

S1.1.1 10x Visium DLPFC

The human dorsolateral prefrontal cortex (DLPFC) 10x Visium dataset (Maynard *et al.*, 2021) is one of the most commonly used benchmarks for spatial domain detection. It comprises 12 postmortem cortical sections, profiled using 10x Genomics Visium platform with paired H&E images. Across these sections, the number of captured spots ranged from 3,460 to 4,789 per slice, with a common gene panel of 33,538 genes. For evaluation, we adopted the manual layer annotations provided in the original study, covering cortical layers 1–6 and white matter (Layer1, Layer2, Layer3, Layer4, Layer5, Layer6 and WM).

S1.1.2 Stereo-seq mouse embryos

The Stereo-seq mouse embryo dataset from Chen *et al.* (2022) includes nine sagittal embryo sections profiled using the high-resolution Stereo-seq platform. Each section comprises 4,356–18,670 spatial locations (bins/spots) and 22,385–25,647 detected genes. We used the embryonic tissue annotations from the original study, including Cavity, Mesenchyme, Connective tissue, Dermomyotome, AGM, Liver, Sclerotome, Brain, Heart, Branchial arch, Neural crest, Notochord, along with several additional minor tissue types.

S1.1.3 MERFISH hypothalamus

To represent high-plex imaging-based ST, we included the MERFISH dataset of the mouse hypothalamic preoptic region from Moffitt *et al.* (2018). This dataset consists of five imaged slices with 5,488–5,926 cells per slice and a targeted panel of 155 genes. We used the anatomical annotations curated in Li and Zhou (2022), which assign cells to major hypothalamic and associated regions, including MPA, MPN, BST, fx, PV, PVH, PVT and V3.

S1.1.4 STARmap cortex

The STARmap dataset from Wang *et al.* (2018) covers mouse medial prefrontal cortex and visual cortex. In total, four slices were used, with 1,049–1,207 cells per slice. Gene coverage varies by experiment: three medial prefrontal cortex slices were profiled with a 166-gene panel, whereas the one visual cortex slice used a 1020-gene panel. We adopted the two levels of manual annotation by Li and Zhou (2022): (i) four anatomical subregions (labels 1–4) for the medial prefrontal cortex data, and (ii) laminar and neighbouring structures for the visual cortex data, including cortical layers (L1, L2/3, L4, L5, L6), corpus callosum (CC), and hippocampus (HPC).

S1.1.5 BaristaSeq primary cortex

As an additional imaging-based targeted-panel dataset, we used the BaristaSeq mouse primary cortex dataset (Long *et al.*, 2023) generated using the BaristaSeq platform (Chen *et al.*, 2018). The dataset comprises three slices with 1,525–2,042 cells per slice and a panel of 79 genes. We used the laminar and white-matter annotations: VISp_I, VISp_II/III, VISp_IV, VISp_V, VISp_VI, VISp_wm, treating each annotated layer or white matter region as a distinct spatial domain.

S1.1.6 Visium HD colorectal cancer (CRC)

To assess SECTOR on a modern high-resolution sequencing-based ST platform, we included a 10x Visium HD human CRC dataset released by Oliveira *et al.* (2025) and annotated in the SACCELERATOR study by Sun *et al.* (2025). We focused on sample P2 CRC, a pretreatment FFPE colorectal adenocarcinoma specimen from the sigmoid colon. Visium HD profiles transcriptome-wide expression on a continuous array of 2 × 2 μm barcoded squares, and the Space Ranger pipeline provides binned outputs at 2-, 8- and 16-μm resolution.

In this study, we converted the Space Ranger filtered feature-barcode matrices and tissue-position files into .h5ad format, matched the corresponding annotations, and analysed the 8- and 16-μm representations of the same tissue section, containing 545,909 and 137,048 bins, respectively, with 18,085 genes. We did not include the 2-μm representation in benchmarking because this sample contains more than eight million bins at that resolution, making fair multi-method comparison impractical at present. Annotations at 8- and 16-μm resolution were obtained from the SACCELERATOR repository and were generated by expert review of the matched H&E image, with marker-gene refinement using genes including EPCAM, VWF and MYL9 (Sun *et al.*, 2025). After removing bins labelled “Outside”, the retained labels were Neoplasm, Connective Tissue, Non-neoplastic Epithelium, Smooth Muscle, Vessel and Veins. These labels were treated as spatial domains for evaluation.

The raw Visium HD CRC dataset is available from 10x Genomics at https://www.10xgenomics.com/platforms/visium/product-family/dataset-human-crc, and the corresponding annotations are available from the SACCELERATOR repository at https://zenodo.org/records/11402686.

S1.1.7 Xenium infiltrating ductal carcinoma (IDC)

To assess SECTOR on a modern imaging-based subcellular-resolution ST platform, we included a 10x Xenium human breast cancer dataset from the Xenium FFPE Human Breast with Custom Add-on Panel resource, curated by Bhuva *et al.* (2024). The matched H&E image was used to assign eight pathology-derived region labels: Invasive Tumour, Stroma, DCIS, Adipose tissue, Cyst, Immune cells, Normal ducts and Blood vessels.

We analysed both the cell-level and hex-bin representations of the IDC sample. After aggregating the original RDS transcript table to cell-level measurements, filtering blank and negative-control probes, retaining labelled cells and converting the result to .h5ad format, the cell-level representation contained 573,091 cells and 380 genes. In parallel, we constructed a hexagonal-bin representation from the same transcript table using 200 hexagons along each axis, following Bhuva *et al.* (2024), and converted it to .h5ad format; this representation contained 30,606 bins and 380 genes. For both representations, we retained only labelled observations and treated the eight pathology-derived labels as spatial domains. Evaluating both the native cell-level data and the coarser hex-bin representation allowed us to assess robustness across spatial granularity on a modern imaging-based platform.

The Xenium IDC dataset curated by Bhuva *et al.* (2024) is accessible from https://zenodo.org/records/10516814.

S1.2 Datasets for case studies

S1.2.1 HER2ST: HER2-positive breast cancer

The HER2ST dataset contains HER2-positive breast cancer samples profiled using 10x Visium (Andersson *et al.*, 2021). In total, 36 slices from eight samples were annotated by pathologists based on H&E staining of the same tissue section. This dataset has been widely used for benchmarking spatial clustering methods (Shang and Zhou, 2022; Yuan *et al.*, 2024) and for evaluating pseudotime/trajectory inference in spatiotemporal methods (Ren *et al.*, 2022; Yu *et al.*, 2025). The raw HER2ST dataset is available at https://github.com/almaan/her2st.

In line with CASCAT (Yu *et al.*, 2025), our study focuses on a single representative slice from sample H1. This slice initially consists of 613 spots and 15,030 genes. Quality control followed the procedure used in previous works (Andersson *et al.*, 2021; Shang and Zhou, 2022), i.e. removing genes with non-zero expression in fewer than 20 spots and spots with fewer than 300 non-zero genes. In addition, we further removed 21 genes associated with a ring pattern observed in multiple samples in the original study (Andersson *et al.*, 2021), as these genes were likely confounded by technical artefacts. After filtering, the H1 slice contained 607 spots and 6,391 genes. We used the pathologist-derived region labels connective tissue, undetermined, cancer in situ, invasive cancer, immune infiltrate, breast glands and adipose tissue both for spatial domain evaluation and as anatomical reference structure for spatiotemporal analyses.

S1.2.2 mouse olfactory bulb (OB)

To further assess the recovery of spatiotemporal organisation in an imaging-based setting, we used a mouse olfactory bulb (OB) dataset derived from a whole-mouse-brain MERFISH atlas (Zhang *et al.*, 2023). The second partition of the atlas, Zhuang-ABCA-2, contains 66 processed slices mapped to the whole mouse brain taxonomy and the Allen CCFv3. Following CASCAT, we focused on a single slice, Zhuang-ABCA-2.005, which has previously been analysed for trajectory reconstruction. This dataset can be downloaded from https://alleninstitute.github.io/abc_atlas_access/descriptions/Zhuang-ABCA-2.html.

This slice originally contains 18,886 cells and 1,122 genes. We excluded cells with completely "unassigned" labels and those annotated as "OLF" (olfactory lobe), resulting in 10,844 cells and 1,122 genes. The remaining cells were annotated into laminar and sublaminar compartments of the main olfactory bulb, including MOBgr (granule layer), MOBmi (mitral cell layer), MOBopl (outer plexiform layer), MOBipl (inner plexiform layer), onl (olfactory nerve layer) and MOB-unassigned (cells in the main olfactory bulb not assigned to a more specific layer). In addition, the slice contains OB cell-type annotations at different developmental stages, including "0167 OB-STR-CTX Inh IMN_2", "0169 OB-STR-CTX Inh IMN_4", "0148 OB Trdn Gaba_1", "0149 OB Trdn Gaba_2", "0161 OB-mi Frmd7 Gaba_1", "0158 OB-out Frmd7 Gaba_1" and "0159 OB-out Frmd7 Gaba_2". We treated the laminar and sublaminar labels as spatial domains and used the OB cell types as an orthogonal reference axis for evaluating recovered trajectories.

S1.3 Summary of dataset sources and evaluation labels

The first five benchmark datasets—10x Visium DLPFC, Stereo-seq mouse embryo, MERFISH hypothalamus, STARmap cortex and BaristaSeq primary cortex—were analysed using the curated versions distributed by SDMBench (Yuan *et al.*, 2024), which have also been adopted in recent method-development and benchmarking studies such as NichePCA (Schaub *et al.*, 2025). SDMBench is available at http://sdmbench.drai.cn/ and https://github.com/zhaofangyuan98/SDMBench.

The Visium HD CRC and Xenium IDC datasets were processed in this study to provide large-scale high-resolution benchmark settings with expert-derived annotations. The HER2ST and mouse OB datasets were used as biologically interpretable case studies for within-section spatiotemporal inference. Across all annotated datasets, benchmarking was restricted to spatial locations with non-missing labels.

S1.4 SECTOR's built-in preprocessing of input datasets

For each dataset with N spatial locations, referring to spots, cells or bins depending on the ST platform, SECTOR started from an expression count matrix and two-dimensional spatial coordinates (x_i, y_i) for each observation. Genes detected in fewer than five spatial locations were removed. Raw counts were then normalised per spatial location to a total of 10,000 counts.

To obtain a compact expression representation, highly variable genes (HVGs) were selected using the Seurat v3 strategy. Unless otherwise stated, the top 2,000 HVGs were retained. For the high-coverage Stereo-seq mouse embryo dataset, we retained 4,000 HVGs. The resulting HVG-filtered expression matrix was scaled to zero mean and unit variance per gene, and principal component analysis (PCA) was used to obtain a d-dimensional feature representation for each spatial location. By default, we used d = 20 principal components; for Stereo-seq, we used d = 50 to better capture its increased transcriptomic complexity.

All SECTOR clustering and pseudotime inference analyses were performed in an unsupervised manner. Manual annotations were used only for external evaluation and biological interpretation, not for model training. When computing supervised metrics, we restricted evaluation to spatial locations with non-missing labels.

S2 Details of spatial and feature graph construction

For each preprocessed dataset of N spatial locations (spots, cells or bins), we start from spatial coordinates (x_i, y_i) for each observation i and a d-dimensional feature matrix $X \in \mathbb{R}^{N\times d}$ obtained by applying principal component analysis (PCA) to the expression of N_{HVG} highly variable genes. SECTOR operates on a graph representation of the ST data in which nodes correspond to spots or cells and edges encode spatial and transcriptional proximity. As illustrated in Fig. 1, we first construct a spatial graph from coordinates and a feature graph from expression, and then combine them to build the fused graph *G*.

S2.1 Spatial graph

The spatial graph captures the physical neighbourhood structure within the tissue. Given spatial coordinates (x_i, y_i) for each node i, we first establish connectivity using a k-nearest neighbour (kNN) approach. Let $\mathcal{N}^{\text{s}}(i)$ denote the set of k_{spatial} closest neighbours to node i under Euclidean distance ($k_{\text{spatial}} = 6$ by default); and let $D_{ij} = \|(x_i, y_i) - (x_j, y_j)\|$ be the Euclidean distance between nodes i and j. We construct the weighted spatial adjacency matrix $A^{\text{s}} \in \mathbb{R}^{N\times N}$ directly using a scale-adaptive Gaussian kernel applied to the symmetric union of these neighbourhoods

$$A_{ij}^{\text{s}} = \begin{cases} \exp(-D_{ij}^2 / 2\sigma^2) & \text{if } j \in \mathcal{N}^{\text{s}}(i) \text{ or } i \in \mathcal{N}^{\text{s}}(j) \text{ or } i = j \\ 0 & \text{otherwise} \end{cases}, \tag{S1}$$

where σ is a global scaling factor set to the median non-zero distance of all edges in the kNN graph. The condition $i = j$ ensures that each node has a self-loop, with $A_{ii}^{\text{s}} = \exp(0) = 1$, facilitating consistent degree computations and graph convolutions.

S2.2 Feature graph

To capture similarity in transcriptional space, we build a feature graph based on a low-dimensional expression embedding E^{f} derived from the PCA feature matrix X via a multilayer perceptron (MLP) with ReLU activation. We establish connectivity using a similar kNN approach in this embedding space. Let $\mathcal{N}^{\text{f}}(i)$ denote the set of k_{feat} nearest neighbours to node i based on cosine similarity ($k_{\text{feat}} = 1$ by default). We construct the weighted feature adjacency matrix $A^{\text{f}} \in \mathbb{R}^{N\times N}$ by averaging the directed neighbourhood weights

$$A_{ij}^{\text{f}} = \frac{w_{ij}^{\text{f}} + w_{ji}^{\text{f}}}{2}, \tag{S2}$$

where the directed weights w_{ij}^{f} represent the cosine similarity for identified neighbours

$$w_{ij}^{\text{f}} = \begin{cases} \dfrac{E_i^{\text{f}} \cdot E_j^{\text{f}}}{\|E_i^{\text{f}}\| \|E_j^{\text{f}}\|} & \text{if } j \in \mathcal{N}^{\text{f}}(i) \\ 0 & \text{otherwise} \end{cases}. \tag{S3}$$

This construction allows the model to connect transcriptionally similar spatial locations even if they are not immediate spatial neighbours, while ensuring the graph remains undirected.

S3 Derivation of two-layer differentiable SE

Here, we derive the two-layer differentiable structural entropy (SE) loss used in SECTOR, starting from the discrete definition provided in Eq. (2) of the main text. For a graph G with adjacency A, the volume (degree) of node i and the total graph volume are defined, respectively, as

$$\deg_i = \sum_{j=1}^{N} A_{ij}, \quad \mathrm{vol}_G = \mathrm{vol}(A) = \sum_{i=1}^{N} \deg_i = \sum_{i,j=1}^{N} A_{ij} . \tag{S4}$$

The SE for a partition tree T over the nodes of G is given by

$$H^T(G) = -\sum_{\alpha \in T \setminus \{r\}} \frac{g_\alpha}{\mathrm{vol}_G} \log \frac{\mathrm{vol}_\alpha}{\mathrm{vol}_{\alpha^{\mathrm{parent}}}} , \tag{S5}$$

where r is the root of T, vol_α is the volume (total degree) of tree node α, $\mathrm{vol}_{\alpha^{\mathrm{parent}}}$ is the volume of the parent of α, and g_α is the cut volume (the total weight of edges from nodes in α to the rest of the graph).

In the context of SECTOR, we establish a fixed two-layer encoding tree T comprising a root r, K cluster nodes ($c_1,...,c_K$), and N leaf nodes ($v_1,...,v_N$) corresponding to individual spatial locations. Consequently, $H^T(G)$ simplifies to a sum over clusters and leaves. Since G is represented by its adjacency matrix A, we subsequently denote the two-layer SE loss as $H(A)$ for simplicity.

Two-layer encoding tree with hard assignments *Y*

In the discrete formulation, each node i is assigned exclusively to a single cluster. We represent this via a one-hot matrix $Y \in \{0,1\}^{N\times K}$ where $Y_{ik} = 1$ if node i belongs to cluster k. The volume of cluster k is then

$$\mathrm{vol}_k = \sum_{i=1}^{N} Y_{ik} \deg_i , \tag{S6}$$

while its internal weight and cut volume are, respectively

$$w_k^{\mathrm{int}} = \sum_{i,j=1}^{N} A_{ij} Y_{ik} Y_{jk}, \quad g_k = \mathrm{vol}_k - w_k^{\mathrm{int}} . \tag{S7}$$

The cut volume of node i is defined as

$$g_i = \deg_i - A_{ii} , \tag{S8}$$

i.e. the sum of weights from node i to all other nodes (excluding the self-loop). The volume of the cluster containing node i (its parent in the tree) is given by

$$\mathrm{vol}_{i^{\mathrm{parent}}} = \sum_{k=1}^{K} Y_{ik} \mathrm{vol}_k . \tag{S9}$$

For this two-layer tree, Eq. (S5) decomposes into contributions from cluster nodes and leaf nodes, i.e.

$$H(A;Y) = -\frac{1}{\mathrm{vol}(A)} \left(\sum_{k=1}^{K} g_k \log \frac{\mathrm{vol}_k}{\mathrm{vol}(A)} + \sum_{i=1}^{N} g_i \log \frac{\deg_i}{\mathrm{vol}_{i^{\mathrm{parent}}}} \right) . \tag{S10}$$

Soft relaxation with assignment matrix *S*

For spatial clustering and pseudotime inference, G corresponds to the undirected fused graph with weighted adjacency matrix $A \in \mathbb{R}^{N\times N}$. To obtain a differentiable objective, we relax the hard assignments Y to a continuous soft assignment matrix $S \in \mathbb{R}^{N\times K}$, such that

$$S_{ik} \geq 0, \quad \sum_{k=1}^{K} S_{ik} = 1 . \tag{S11}$$

Here, S_{ik} is interpreted as the probability that node i belongs to cluster k. The cluster volume becomes an expected soft volume

$$\mathrm{vol}_k = \sum_{i=1}^{N} S_{ik} \deg_i , \tag{S12}$$

and the internal weight and cut volume of cluster k are derived as

$$w_k^{\mathrm{int}} = \sum_{i,j=1}^{N} A_{ij} S_{ik} S_{jk}, \quad g_k = \mathrm{vol}_k - w_k^{\mathrm{int}} . \tag{S13}$$

The node-level cut volume g_i remains as defined in Eq. (S8). Under soft assignments, we approximate the parent volume by the expected cluster volume under the distribution S_i

$$\mathrm{vol}_{i^{\mathrm{parent}}} = \sum_{k=1}^{K} S_{ik} \mathrm{vol}_k . \tag{S14}$$

Substituting Eq. (S4), Eq. (S8) and Eqs. (S11)–(S14) into Eq. (S10) yields the differentiable two-layer SE objective

$$H(A;S) = -\frac{1}{\mathrm{vol}(A)}(\sum_{k=1}^{K} g_k \log\frac{\mathrm{vol}_k}{\mathrm{vol}(A)} + \sum_{i=1}^{N} g_i \log\frac{\deg_i}{\mathrm{vol}_{i^{\text{parent}}}})$$

$$= -\frac{1}{\sum_{i,j=1}^{N} A_{ij}}\left(\sum_{k=1}^{K}\left(\sum_{i=1}^{N} S_{ik}\sum_{j=1}^{N} A_{ij} - \sum_{i,j=1}^{N} A_{ij}S_{ik}S_{jk}\right)\log\frac{\sum_{i=1}^{N} S_{ik}\sum_{j=1}^{N} A_{ij}}{\sum_{i,j=1}^{N} A_{ij}} + \sum_{i=1}^{N}\left(\sum_{j=1}^{N} A_{ij} - A_{ii}\right)\log\frac{\sum_{j=1}^{N} A_{ij}}{\sum_{k=1}^{K} S_{ik}\sum_{i=1}^{N} S_{ik}\sum_{j=1}^{N} A_{ij}}\right), \tag{S15}$$

which corresponds to Eq. (3) in the main text. The first term quantifies the information cost of transitions between clusters, while the second term quantifies transitions from clusters to individual nodes. Since all terms in Eq. (S15) are smooth in S for a fixed fused adjacency matrix A, $H(A;S)$ is differentiable with respect to the neural network parameters that produce S. In SECTOR, the kNN-based construction of the feature graph is treated as a graph-construction step rather than as a fully differentiable neighbour-selection operation.

Minimising $H(A;S)$ therefore encourages encodings in which random walks rarely cross domain boundaries, favouring clusters that are internally well-connected and externally well-separated (i.e. possessing small boundary volume).

S4 Details of SECTOR implementation and parametrisation

S4.1 Training protocol and hyperparameters

As described in Supplementary Section S1.4, we retained 2,000 HVGs for most datasets and 4,000 HVGs for the Stereo-seq dataset, based on the sensitivity analysis. The PCA dimension was set to $d = 20$ for most datasets and $d = 50$ for Stereo-seq to better capture its greater transcriptomic complexity. We train SECTOR for at most epochs = 1000 iterations using the Adam optimiser with learning rate 1×10^{-3}. The embedding dimension of both the MLP and GCN layers is $d_{\text{emb}} = 64$, with ReLU activations and without dropout. The spatial adjacency matrix A^{s} and the feature adjacency matrix A^{f} are derived from k-nearest neighbours (kNN) graphs with default $k_{\text{spatial}} = 6$ (except for very large datasets like Visium HD CRC 2-μm binned output or Xenium IDC cell-level section we recommend $k_{\text{spatial}} = 12$) and $k_{\text{feat}} = 1$ neighbours (except for Stereo-seq we recommend $k_{\text{feat}} = 3$), respectively. The two matrices are combined into a fused adjacency matrix A with $\beta = 0.5$ by default; this parameter down-weights the contribution of A^{f} relative to A^{s}.

The structural entropy (SE) objective is optimised jointly with a spatial total variation (TV) term and, when active, the balance regulariser. The TV coefficient λ is warmed up from 0 to its target value λ_{TV} over 100 epochs using a linear schedule

$$\lambda_{\text{TV}}^{(e)} = \lambda_{\text{TV}} \max\{0, \min(1, \frac{e - e_{\text{start}}}{e_{\text{end}} - e_{\text{start}}})\}, \tag{S16}$$

where e is the current epoch; e_{start} and e_{end} denote the start and end epochs of the warm-up window. This schedule allows SE to first organise coarse spatial domains before strong spatial smoothing is imposed. Based on parameter sensitivity analysis (Fig. 4), we used the following default values: λ_{TV}=2.0 for 10x Visium, MERFISH and BaristaSeq, λ_{TV}=3.0 for Stereo-seq and λ_{TV}=4.0 for STARmap, Visium HD and Xenium. All models are trained on the full graph without mini-batches.

S4.2 Label-free diagnostics and early stopping

To avoid over-fitting and to automatically detect convergence in the unsupervised setting, SECTOR monitors several label-free graph diagnostics during training.

S4.2.1 Spatial structural entropy

We compute a spatial SE score H_{spatial} that mirrors the differentiable SE loss but is evaluated only on the spatial adjacency matrix A^{s}. Concretely, H_{spatial} is the two-layer SE of A^{s} with respect to the current soft assignments S. Lower values of H_{spatial} correspond to more spatially coherent domains with smaller boundary volume.

S4.2.2 Edge-agreement scores

We also track how consistent cluster labels are along the spatial edges. For each non-self edge (i, j) in A^{s}, we define:

- Soft edge agreement (EAS_{soft}): the weighted mean similarity of the assignment distributions

$$\text{EAS}_{\text{soft}} = \frac{\sum_{i,j} A_{ij}^{\text{s}} \sum_{k=1}^{K} S_{ik}S_{jk}}{\sum_{i,j} A_{ij}^{\text{s}}}. \tag{S17}$$

- Hard edge agreement (EAS_{hard}): the weighted fraction of edges whose endpoints share the same hard label $\hat{y}$

$$\text{EAS}_{\text{hard}} = \frac{\sum_{i,j:\hat{y}_i=\hat{y}_j} A_{ij}^{\text{s}}}{\sum_{i,j} A_{ij}^{\text{s}}}. \tag{S18}$$

Both scores lie in [0,1]; larger values indicate smoother, more spatially consistent domains. In practice, we use $\mathrm{EAS}_{\mathrm{soft}}$ in the early-stopping criterion, while $\mathrm{EAS}_{\mathrm{hard}}$ is monitored as an additional diagnostic.

S4.2.3 Early stopping strategy

Early stopping combines improvement in the unsupervised metrics with stability of the assignments. At each training epoch, SECTOR performs the following steps:

(1) Update best unsupervised state

We track the best values of H_{spatial} and $\mathrm{EAS}_{\mathrm{soft}}$ observed so far. An epoch is considered an improvement if H_{spatial} decreases by at least a relative tolerance (default 0.005) or $\mathrm{EAS}_{\mathrm{soft}}$ increases by at least the same relative tolerance. When an improvement occurs, we reset the unsupervised "no-improvement" counter. If the improvement comes from H_{spatial}, we also record the current hard labels as the best unsupervised state, and optionally save a model checkpoint. If no improvement occurs, we increment the no-improvement counter.

(2) Monitor stability

We maintain a sliding window (default length 4) of the number of used clusters K_{used}. Assignments are considered stable if K_{used} is constant over this window, and the self-NMI between the current hard labels and those from the previous epoch exceeds a threshold (default 0.97). Each time both conditions hold, we increment a "stability hit" counter; otherwise, we reset it to zero. By default, at least three such stability hits are required.

(3) Stopping rule

Training stops early when there has been no improvement in either H_{spatial} or $\mathrm{EAS}_{\mathrm{soft}}$ for six consecutive checks and the stability-hit counter has reached its required value (default 3).

The final clustering used for evaluation and downstream analysis is the unsupervised state corresponding to the lowest H_{spatial} encountered during training, rather than the last training epoch.

S4.3 Post hoc processing

After training, we apply an optional post-hoc "island" cleanup step on the spatial adjacency matrix A^s to remove tiny disconnected fragments. This step operates only on the final hard labels $\hat{y}$ and does not change the soft assignments *S*. For each cluster label *c*, we consider the induced subgraph containing only nodes with this label, and compute its connected components and their sizes. Let $n_{\max}$ be the size of the largest component for label *c*. A component is marked as an island if its size is less than $\varepsilon_{\mathrm{abs}}$ or less than $\varepsilon_{\mathrm{frac}} \cdot n_{\max}$. By default, we use dataset-specific thresholds: for Visium slices we set $\varepsilon_{\mathrm{abs}}=40$ and $\varepsilon_{\mathrm{frac}}=0.1$; for Stereo-seq slices we disable island cleanup due to complex embryonic annotations of this dataset; for other datasets such as MERFISH, STARmap, BaristaSeq, Visium HD, and Xenium we use $\varepsilon_{\mathrm{abs}}=20$ and $\varepsilon_{\mathrm{frac}}=0.03$. For each node in an island, we inspect all spatial neighbours and reassign the node to the neighbour label with the largest accumulated boundary weight (the sum of edge weights to neighbours with that label). This procedure is repeated for up to two passes or until no changes occur. The effect is to remove small, visually implausible islands while leaving larger regions essentially unchanged.

S4.4 Cluster-balance regulariser and balance probe

Minimising SE encourages partitions that reduce the total weight of edges crossing between clusters while increasing the volume of well-supported clusters. Because empty or very small clusters contribute little to the entropy, the objective does not by itself require all *K* clusters to be used evenly. In practice, SE-based models can therefore reduce the loss by allowing a small subset of clusters to absorb most nodes while leaving other clusters under-used or effectively empty. A related tendency has been observed in other deep structural-entropy frameworks such as DeSE (Zhang *et al.*, 2025), where smaller ground-truth clusters may be merged into larger ones or split across them, and extra cluster prototypes can remain unused when the specified cluster number exceeds the structure supported by the data.

In ST data, severe early under-use of clusters can reduce the resolution of small but meaningful tissue domains when K is chosen to reflect expected anatomical or pathological structures. Tissues often contain both large domains, such as broad cortical layers or stromal regions, and smaller domains, such as thin laminae, germinal niches or small subnuclei. If optimisation is driven only by SE and spatial TV, small but genuine regions may be absorbed into neighbouring large domains because they occupy relatively few spatial locations. SECTOR therefore includes an optional cluster-balance regulariser and a short balance probe as a safeguard against severe cluster collapse, while still allowing unequal domain sizes and data-supported coarser partitions.

S4.4.1 Balance regulariser

To discourage the behaviour of assigning most spatial locations into a small subset of clusters, we include an optional cluster-balance penalty. Let $S \in \mathbb{R}^{N \times K}$ denote the soft assignment matrix with *K* clusters and let $\deg_i$ be the degree of node *i* in the fused graph *G* represented by the fused adjacency matrix *A*. The empirical cluster-usage distribution $p \in \mathbb{R}^K$ is defined either in "volume" or "node" mode

- Volume mode (default for sequencing-based ST data)

$$p_k = \frac{\sum_{i=1}^{N} S_{ik} \deg_i}{\sum_{i=1}^{N} \deg_i}, \qquad k = 1, ..., K. \tag{S19}$$

- Node mode (default for imaging-based ST data)

$$p_k = \frac{1}{N} \sum_{i=1}^{N} S_{ik}. \tag{S20}$$

The target usage $u \in \mathbb{R}^K$ is uniform by default, i.e. $u_k = 1/K$. The balance loss is the Kullback-Leibler (KL) divergence between p and u

$$\mathcal{L}_{\text{bal}}(S) = \sum_{k=1}^{K} p_k \log \frac{p_k + \varepsilon}{u_k + \varepsilon}, \tag{S21}$$

with a small numerical constant ε to avoid log-zeros. The full training loss is then

$$\mathcal{L}_{\text{total}} = H(A;S) + \lambda \text{TV}(S) + \gamma \mathcal{L}_{\text{bal}}(S), \tag{S22}$$

where γ is the balance weight (default $\gamma = 1.0$ for sequencing-based data and $\gamma = 0.1$ for imaging-based data). When the balance prior is inactive, we set $\gamma = 0$, in which case $\mathcal{L}_{\text{bal}}(S)$ is effectively ignored.

S4.4.2 Balance probe

By default, SECTOR does not enable the balance prior immediately. Instead, it runs a short "balance probe" to check whether the model naturally uses the expected number of clusters *K* without explicit balancing.

(1) Probe phase
For a number of balance-probe epochs (default 20) we train with γ=0 (balance off), using the same learning rate and TV warm-up schedule as in the main training loop.

(2) Monitoring cluster usage
At each probe epoch we compute the number of used clusters K_{used}, i.e. the number of clusters whose hard assignment contains at least one spot or cell. During the last 10% of probe epochs, we count a "stable hit" whenever K_{used} equals *K*.

(3) Decision
If the number of stable hits equals the number of epochs in this last 10% window, we consider the probe successful and continue training with the balance prior disabled (γ=0). Otherwise, we restart the model from a fresh random initialisation, enable the balance term ($\gamma > 0$), and then train for the full number of epochs.

This procedure ensures that the balance prior is only used when needed (e.g. for highly imbalanced or noisy datasets) and leaves the main SE + TV objective unchanged when it already yields well-distributed clusters.

S4.4.3 Guidance for setting the balance probe and weight

The cluster-balance prior in SECTOR is intended as a safeguard against under-use of clusters, rather than as a routine tuning parameter. In particular, its purpose is to prevent the SE + TV objective from assigning most spatial locations to only a small subset of clusters while leaving the remaining clusters under-used or empty. Accordingly, users should normally treat the balance probe as the first decision step and use the balance weight γ only when the probe indicates that explicit balancing is necessary.

Default workflow

We recommend keeping the balance probe enabled. During the probe phase, SECTOR is trained with γ=0 for a short number of epochs and monitors the number of used clusters K_{used}. If all *K* clusters are stably occupied by the end of the probe, then the main training run should continue with the balance prior disabled, that is, γ=0. If the probe fails, training should be restarted with the balance term activated. By default, SECTOR uses volume mode with γ=1.0 for sequencing-based ST data and node mode with γ=0.1 for imaging-based ST data. These defaults reflect the different granularity of the two data types: sequencing-based data are naturally weighted by graph volume, whereas imaging-based data are more naturally balanced by the number of cells.

How to interpret γ

The balance weight should be interpreted as the strength of a weak occupancy prior rather than as a target that forces all domains to have the same size. Small values of γ leave the SE + TV objective largely unchanged, while larger values increasingly discourage empty or severely under-used clusters. In practice, users should aim to use the smallest γ that restores stable use of all *K* clusters when cluster under-use occurs. If a modest γ already yields stable occupancy, increasing it further is usually unnecessary.

When to increase γ

A larger γ may be helpful if, after restart, one or more clusters remain effectively empty, or if K_{used} continues to be substantially smaller than *K* for much of training. In such cases, γ can be increased gradually, for example in small multiplicative steps, until all clusters are used stably. This situation is more likely to arise in noisy datasets, in highly imbalanced tissues, or when the user-specified *K* is close to the upper end of what the data can support.

When to decrease γ

If the inferred domains become unnaturally equal-sized, if large biological regions are split unnecessarily, or if the model appears to over-fragment smooth tissue structure, then γ is likely too strong and should be reduced. This is particularly relevant for tissues that are expected to contain highly unequal domain sizes, where a strong balance prior may over-correct a real biological imbalance.

When to reconsider *K*

If the balance probe repeatedly fails and under-use of clusters persists even after increasing γ to a moderate level, then the issue may not be insufficient balancing but rather an overly large user-specified number of clusters. In this case, users should reconsider K itself instead of continuing to strengthen the balance prior.

Guidance on probe length

The default probe length is usually adequate, but longer probes may be beneficial for especially large or noisy datasets, or when cluster usage fluctuates strongly near the end of the probe window. In such cases, increasing the number of probe epochs can make the decision more reliable.

In summary, the recommended practical strategy is: (i) keep the balance probe enabled; (ii) use $\gamma=0$ if the probe succeeds; (iii) if the probe fails, restart with the built-in default γ for the corresponding data type; and (iv) adjust γ only if cluster under-use persists or if balancing becomes visibly too strong. Under this strategy, the balance prior acts as a targeted rescue mechanism rather than a standard optimisation component.

S4.5 Sparse large-scale implementation for high-resolution ST datasets

To enable SECTOR to run on large-scale high-resolution ST datasets such as Visium HD and Xenium, we implemented a scale-aware sparse mode. This implementation preserves the same SECTOR objective, including the differentiable structural-entropy loss, spatial TV regularisation and optional balance regularisation, but avoids dense $N\times N$ distance or adjacency matrices when the number of spatial locations N is large.

By default, SECTOR selects the implementation according to dataset size. For datasets with $N<10{,}000$, the original dense implementation is used. For intermediate-scale datasets ($10{,}000\le N<T$, where $T=100{,}000$ by default), SECTOR switches to sparse graph construction while retaining an MLP-derived feature graph. For very large datasets ($N\ge T$), SECTOR uses sparse graph construction and builds the feature graph directly from the PCA feature representation, rather than repeatedly reconstructing it from a changing neural representation. Spatial and feature graphs are constructed as sparse nearest-neighbour graphs and stored as sparse tensors, avoiding full pairwise distance or dense adjacency matrices. During training, SECTOR operates on sparse graph edges and edge weights.

This sparse large-scale implementation reduces memory usage and makes training practical for datasets with hundreds of thousands of spatial locations. It is an implementation-level scalability adaptation and does not change the core SECTOR objective. In our study, this implementation was used for the large-scale Visium HD CRC and Xenium IDC benchmarks.

S4.6 Pseudotime orientation

The spectral pseudotime is defined only up to an overall sign and affine transformation. SECTOR therefore provides two mechanisms to choose a biologically meaningful direction.

(1) Spatial anchor

Users can specify a spatial anchor direction ("north", "south", "east" or "west") based on the physical coordinates (x_i, y_i). For example, a "south" anchor selects the n_{anchor} spatial locations with the largest y-coordinate, whereas a "north" anchor selects those with the smallest y; analogously, "west" and "east" use the smallest and largest x-coordinates, respectively. Let R_{anchor} be the set of indices corresponding to the selected anchor spatial locations. We flip the sign of the pseudotime vector t if the mean pseudotime over R_{anchor} is larger than the global mean, so that the anchor region is assigned low pseudotime values. By default, we use $n_{\text{anchor}}=200$ or all spatial locations if $N<200$.

(2) Root cluster

Alternatively, if a "root" cluster is specified (e.g. a known progenitor zone), we orient pseudotime so that this cluster has low values. Let R_{root} be the set of indices belonging to the root cluster. As above, we flip the sign of t if the mean pseudotime over R_{root} exceeds the global mean, ensuring that the root cluster lies at the beginning of the trajectory.

In both cases, the oriented pseudotime is then min–max scaled to the interval $[0,1]$. These two options (root cluster or spatial anchor) provide flexible, dataset-specific control over the biological interpretation of the inferred pseudo-temporal axis while leaving the underlying spectral ordering unchanged.

S5 Benchmarking and case-study analysis details

S5.1 Spatial clustering benchmarking setup and evaluation metrics

We evaluated SECTOR along two axes: spatial-domain detection and within-section pseudotime recovery. For spatial-domain benchmarking, we used the seven benchmark datasets described in Supplementary Section S1 and compared SECTOR with spatiotemporal methods that jointly perform spatial clustering and pseudotime inference, as well as spatial clustering-focused methods. Clustering accuracy was evaluated using normalised mutual information (NMI), homogeneity (HOM) and completeness (COM), following Yuan *et al.* (2024). NMI measures overall agreement between predicted and annotated domains, whereas HOM and COM respectively quantify cluster purity within ground-truth regions and fragmentation of ground-truth regions across predicted clusters.

The spatiotemporal baselines were CASCAT, stLearn and SpaceFlow. The spatial clustering-focused baselines run in this study were NichePCA, SpaSEG, CellCharter, MENDER, NicheCompass, BANKSY and GraphST. CellCharter, MENDER, NicheCompass, BANKSY and GraphST were included as strong contemporary baselines highlighted by Dong *et al.* (2025), while NichePCA and SpaSEG were included to represent recent clustering-focused advances. For the large-scale high-resolution ST group, comparisons were restricted to methods that were feasible under the computational resources used in this study. In addition, for the standard benchmark datasets shared with SDMBench, we report an extended comparison with earlier but widely used spatial clustering methods, including SpaGCN, SCAN-IT, STAGATE, CCST, SEDR, BayesSpace and BASS, using the released per-slice metrics from Yuan *et al.* (2024).

For method parameterisation, we followed the strategy described in the main Methods. When dataset-specific recommended settings were provided by the original authors, we used those settings directly; otherwise, we searched within the recommended parameter space to identify a strong configuration. When paired histology images were unavailable or image-aware processing was impractical at scale, stLearn was run in its expression-plus-spatial-distance configuration.

For methods run in this study, we followed Yuan *et al.* (2024) by repeating each method–slice combination ten times using the same parameter settings, with different random initialisations where applicable. For each slice, we used the median value across the ten runs as the per-slice NMI, HOM and COM value. These per-slice median values were used to generate the boxplots in Fig. 2 and, for the primary methods, the extended comparison in Supplementary Fig. S4; earlier baselines in Supplementary Fig. S4 used the released per-slice metrics from Yuan *et al.* (2024). Repeated runs were used to reduce stochastic variability, not to increase the number of independent slice-level observations. All methods run in this study were executed on a Linux server with 100 CPU cores, 500 GB RAM and one NVIDIA L40 GPU with 48 GB memory.

S5.2 Runtime, memory and controlled scaling analysis

For computational-efficiency comparisons, we recorded runtime and peak memory usage for SECTOR and the spatiotemporal baselines. Values were summarised at the dataset level for the benchmark datasets shown in Fig. 4b.

To reduce confounding from platform and tissue context in the scaling analysis, we performed a controlled within-sample analysis using the Visium HD CRC dataset. Spatially contiguous central subsets were generated from the 16-μm binned output by selecting tissue windows of increasing size. Each larger subset contained the smaller subset, preserving local neighbourhood structure while increasing the number of spatial locations. This design avoids random subsampling, which can disrupt graph structure and confound runtime or memory comparisons involving graph construction. The full 8-μm binned output was additionally included to extend the scaling range to 545,909 bins.

S5.3 Case-study evaluation of within-section pseudotime

We used the HER2ST breast cancer and mouse OB datasets to evaluate whether inferred pseudotime captured biologically interpretable within-section progression patterns. For both case studies, SECTOR was compared with CASCAT, stLearn and SpaceFlow using clustering accuracy and pseudotime smoothness. Because spatial pseudotime lacks a direct ground-truth ordering, we further supported the interpretation with downstream computational analyses: pseudotime–gene correlation and Gene Ontology (GO) enrichment for HER2ST, and supertype-resolved UMAP projection and marker-gene profiling for mouse OB.

S5.3.1 Evaluation of pseudotime smoothness by spatial semivariance

Alongside clustering accuracy comparisons with CASCAT, stLearn and SpaceFlow, we evaluated the smoothness of the inferred pseudotime fields using a spatial statistics metric, the empirical semivariance. For each method, we computed the semivariogram with the classical Matheron estimator, following the formulation of Cressie and Hawkins (1980) and its recent use in stLearn (Pham *et al.*, 2023), i.e.

$$\nu(h) = \frac{1}{2|\mathcal{N}(h)|} \sum_{(i,j)\in\mathcal{N}(h)} (t_i - t_j)^2 , \qquad \text{(S23)}$$

where t_i and t_j are pseudotime values at spatial locations i and j, and $\mathcal{N}(h)$ is pairs of spatial locations whose Euclidean distance falls within a given distance-lag bin centred at h. To make semivariance curves comparable across methods with different pseudotime scales, $\nu(h)$ was normalised by the sample variance of the corresponding pseudotime vector, yielding a unitless relative semivariance.

S5.3.2 Transition-gene and gene-ontology (GO) analysis of HER2ST

For the HER2ST case study, downstream analysis focused on spots assigned to predicted domains corresponding to the main cancer-associated progression branch, namely breast glands, cancer in situ and invasive cancer. For SECTOR and each baseline method, this subset was defined by matching predicted domains to the same three annotated tissue regions.

Within this subset, gene–pseudotime associations were computed using Spearman correlation (ρ) between processed gene expression and the inferred pseudotime, implemented with scipy.stats.spearmanr. The analysis was performed on the processed expression matrix after feature selection. If adata.var["highly_variable"] was available, those genes were used; otherwise, highly variable genes were computed with scanpy.pp.highly_variable_genes using the Seurat v3 flavour, retaining the top 2,000 HVGs by default. Then, p values were adjusted across genes using the Benjamini–Hochberg procedure implemented in statsmodels.stats.multitest.multipletests with method = "fdr_bh".

A mirrored bar plot of the top correlated genes (transition genes) was generated with eval_pt.transition_markers_plot_global in the SECTOR Python package; this function was implemented following the plotting strategy used in stLearn (Pham *et al.*, 2023). Positive and negative transition genes were defined as genes with adjusted $p < 0.05$ and $\rho > 0.3$ or $\rho < -0.3$, respectively. When more than 100 genes passed this filter in a given direction, the top 100 ranked by $|\rho|$ were used for enrichment to keep gene-list sizes comparable. GO enrichment was performed separately for the positive and negative transition-gene lists using GSEApy (gseapy.enrichr) with organism="Human" and cutoff=0.05. The GO library for enrichment analysis was selected by gseapy.get_library_name(), with the latest matching GO Biological Process library used at runtime ("GO_Biological_Process_2025"). Enrichment results from the positive and negative gene lists were concatenated, restricted to terms with adjusted $p < 0.05$. The combined enrichment table was plotted by ranking terms by adjusted p values and displays the top 10 terms as horizontal bars using $-\log_{10}$(adjusted p values). This overall strategy is aligned with stLearn (Pham *et al.*, 2023), which also identifies transition genes by Spearman correlation with inferred pseudotime before pathway analysis.

S5.3.3 UMAP projection and marker-gene analysis of mouse OB

For the mouse OB case study on the MERFISH Zhuang-ABCA-2.005 slice, we focused on seven annotated OB inhibitory-neuron supertypes on the maturation axis: 0167 OB-STR-CTX Inh IMN_2, 0169 OB-STR-CTX Inh IMN_4, 0148 OB Trdn Gaba_1, 0149 OB Trdn Gaba_2, 0161 OB-mi Frmd7 Gaba_1, 0158 OB-out Frmd7 Gaba_1, and 0159 OB-out Frmd7 Gaba_2. For UMAP visualisation, we first subset the data to the seven OB supertypes, then computed a k-nearest-neighbor graph in Scanpy using sc.pp.neighbors with n_neighbors = 10. For each method (SECTOR, CASCAT, stLearn and SpaceFlow), the graph was built from the corresponding learned embeddings, e.g. use_rep = "sector_embedding" for SECTOR. UMAP coordinates were then computed with sc.tl.umap, and the resulting embedding was visualised with sc.pl.umap, colouring cells either by supertype or by the corresponding method-specific pseudotime, e.g. "pseudotime" for SECTOR, with pseudotime displayed on a fixed range of 0–1. This evaluation strategy is consistent with CASCAT (Yu *et al.*, 2025), which on the same Zhuang-ABCA-2.005 dataset used supertype-resolved UMAP and marker-gene analyses to assess OB maturation trajectories.

For downstream marker analysis, the raw expression matrix was normalised with Scanpy using sc.pp.normalize_total (target_sum = 1e4) and log-transformed with sc.pp.log1p. Differential expression between each supertype and the rest of supertypes was performed with sc.tl.rank_genes_groups using method = "wilcoxon" and corr_method = "benjamini-hochberg". Genes were retained if adjusted $p < 0.05$ and log fold-change > 0.5. For each supertype, the top five marker genes were then selected by descending scores and log fold-changes. To visualise how these developmental markers were distributed across inferred spatial domains, we reordered the predicted regions of each method, e.g. the categorical sequence [5, 1, 2, 0, 3, 4] for SECTOR, and plotted a Scanpy dot plot with sc.pl.dotplot. This plot was used to assess whether the spatial domains (inferred by each method) aligned with the expected immature-to-mature OB neuronal program.

S6 Comparison of HVG- and SVG-based feature graph construction

In SECTOR, the feature graph is constructed from a PCA representation of selected genes. In the default pipeline, these genes are chosen as HVGs, and the resulting PCA feature matrix is used to build the expression-based adjacency matrix that is fused with the spatial graph. Because spatially variable genes (SVGs) may be more directly associated with tissue architecture, we examined whether replacing HVGs with SVGs would improve SECTOR. This comparison was additionally motivated by related work such as Chrysalis (Túrós *et al.*, 2024), which first identifies SVGs using Moran's I and then applies PCA to the SVG matrix for downstream spatial analysis.

To evaluate this design choice, we implemented an SVG-based variant of SECTOR. Specifically, the default HVG-selection step used to generate the PCA feature matrix was replaced by SVG ranking based on Moran's I, following the SVG-detection strategy described in Chrysalis. The resulting SVG matrix was then used to compute the PCA feature matrix that serves as input to SECTOR for feature-graph construction. All other components and parameters of SECTOR were kept unchanged.

We compared the default HVG-based and SVG-based variants on three representative datasets spanning different technologies and scales: 10x Visium DLPFC slice 151673, Stereo-seq mouse embryo slice E9.5_E1S1.MOSTA, and the Visium HD CRC tissue section at 16-µm bin resolution (Supplementary Fig. S7). Across all three examples, the HVG-based design consistently achieved higher clustering accuracy than the SVG-based alternative. On DLPFC, the NMI decreased from 0.7126 with HVGs to 0.6565 with SVGs. On Stereo-seq, the NMI decreased from 0.5819 to 0.5435. On Visium HD, the NMI decreased from 0.6991 to 0.6424. Qualitatively, both variants recovered the broad tissue architecture, but the HVG-based version yielded cleaner spatial boundaries and more stable spatial domains. The pseudotime fields were broadly similar for DLPFC and Stereo-seq, whereas on Visium HD the SVG-based variant produced a more distorted field concentrated around a narrow tissue interface, suggesting reduced robustness of the inferred gradient.

These results indicate that, although SVGs are a reasonable alternative design choice, they did not improve SECTOR in the representative settings examined here. A plausible explanation is that SECTOR already incorporates spatial information explicitly through the fused spatial–expression graph and spatial total variation regularisation. In this context, restricting the expression embedding to SVGs may overemphasise genes with strong spatial autocorrelation while excluding non-spatial, yet still domain-discriminative, transcriptomic variation that helps distinguish tissue compartments. We therefore retain HVG-based PCA as the default feature-construction strategy in SECTOR.

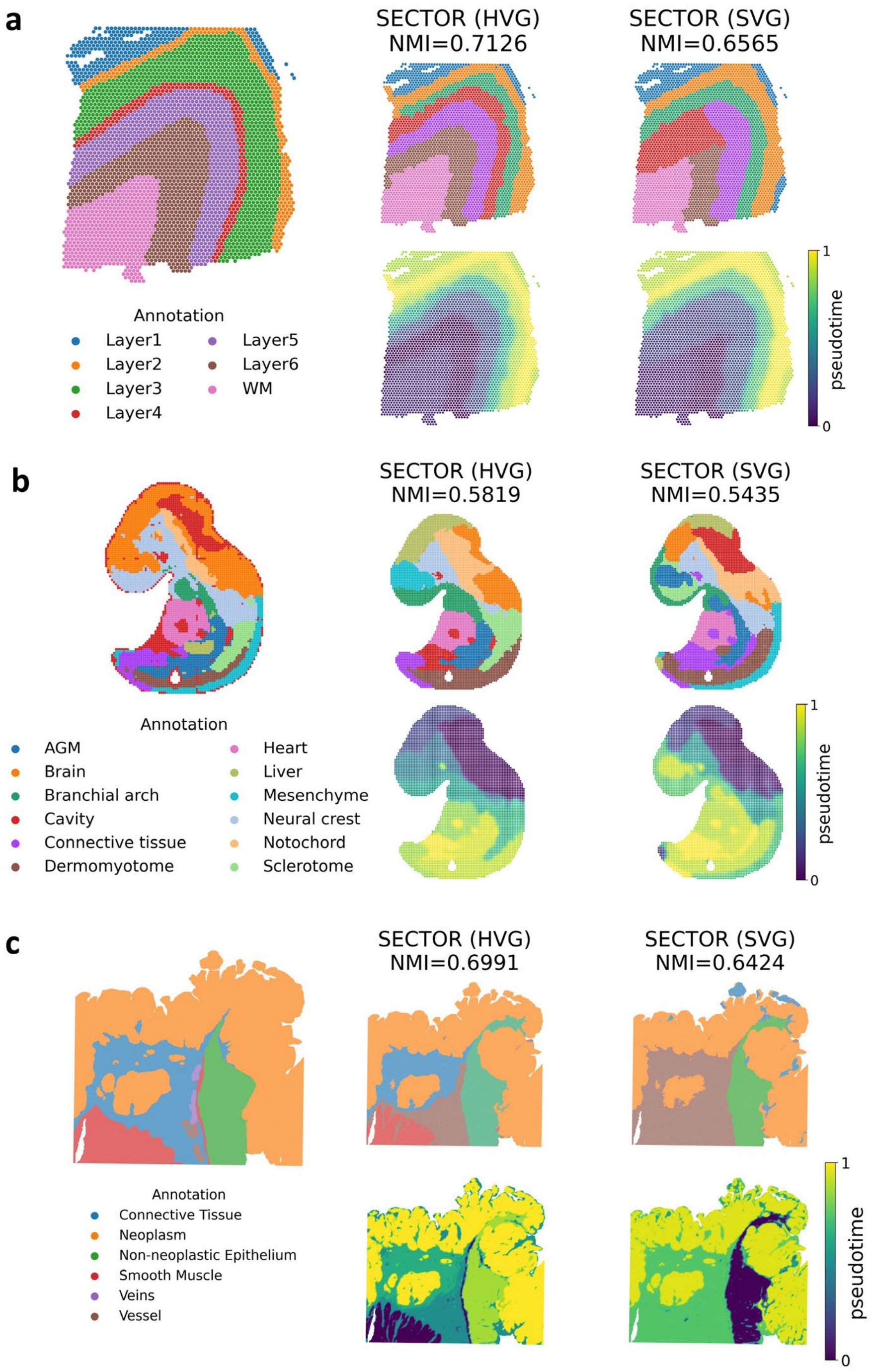

Figure S7. Comparison of HVG- and SVG-based feature graph construction in SECTOR. For each representative slice, the left panel shows the ground-truth annotation, the middle panel shows SECTOR using a PCA feature matrix constructed from highly variable genes (HVGs), and the right panel shows SECTOR using a PCA feature matrix constructed from spatially variable genes (SVGs). SVGs were selected using a Moran's *I*-based ranking following the strategy described in Chrysalis (Túrós *et al.*, 2024), while all other components of SECTOR were kept unchanged. The upper plot in each method panel shows the inferred spatial domains together with NMI against the annotation, and the lower plot shows the inferred pseudotime field. (**a**) 10x Visium human DLPFC slice 151673. (**b**) Stereo-seq mouse embryo slice E9.5_E1S1.MOSTA. (**c**) Visium HD CRC slice at 16-μm bin resolution.

S7 Sensitivity analysis of gene panel size within the Visium HD CRC 16-μm binned output

To isolate sensitivity to feature density without confounding from tissue type or sequencing platform, we performed a within-dataset gene down-sampling analysis on the single Visium HD CRC 16-μm binned output containing 137,048 bins and 18,085 genes. Starting from the full processed feature set, we progressively restricted the number of genes used for modelling and evaluated SECTOR using the same annotations and fixed model hyperparameters. Specifically, we computed one global HVG ranking on the full transcriptome (18,085 genes) for the Visium HD CRC 16-μm binned output using scanpy.pp.highly_variable_genes with flavor="seurat_v3", and then formed nested panels of 18,085 (full), 10,000, 6,000, 3,000, 1,500, 1,000, 500 and 250 genes.

The results are shown in Supplementary Fig. S8. Overall, SECTOR was reasonably robust to moderate reductions in feature density. The full feature set achieved the best clustering accuracy (NMI = 0.6991), but performance remained close at 3,000 genes (NMI = 0.6761) and 1,500 genes (NMI = 0.6680), with the inferred spatial domains and pseudotime patterns closely matching the overall tissue architecture. At 1,000 genes, SECTOR still recovered the major tissue compartments (NMI = 0.6366), although with loss of boundary precision. A more noticeable degradation appeared only for very small panels, especially at 500 genes (NMI = 0.5899) and 250 genes (NMI = 0.5768), where the inferred domains became less stable and the pseudotime field showed reduced spatial coherence.

Interestingly, performance was not strictly monotonic among the larger reduced panels: the 3,000- and 1,500-gene panels performed better than the 10,000- and 6,000-gene panels. This suggests that, at least for this dataset, a compact set of highly informative genes can be sufficient for SECTOR and that simply increasing panel size does not necessarily improve domain detection once many lower-priority features are included.

Taken together, this analysis implies that SECTOR is not highly sensitive to moderate reductions in gene density when informative genes are retained, and that panels on the order of approximately 1,500–3,000 genes may still support reliable spatial domain detection in this setting. However, because the reduced panels here were generated from the top-ranked HVGs of the same whole-transcriptome dataset, this should be viewed as an informative best-case scenario; in practice, performance will also depend on how well a targeted panel captures the biology relevant to the tissue under study.

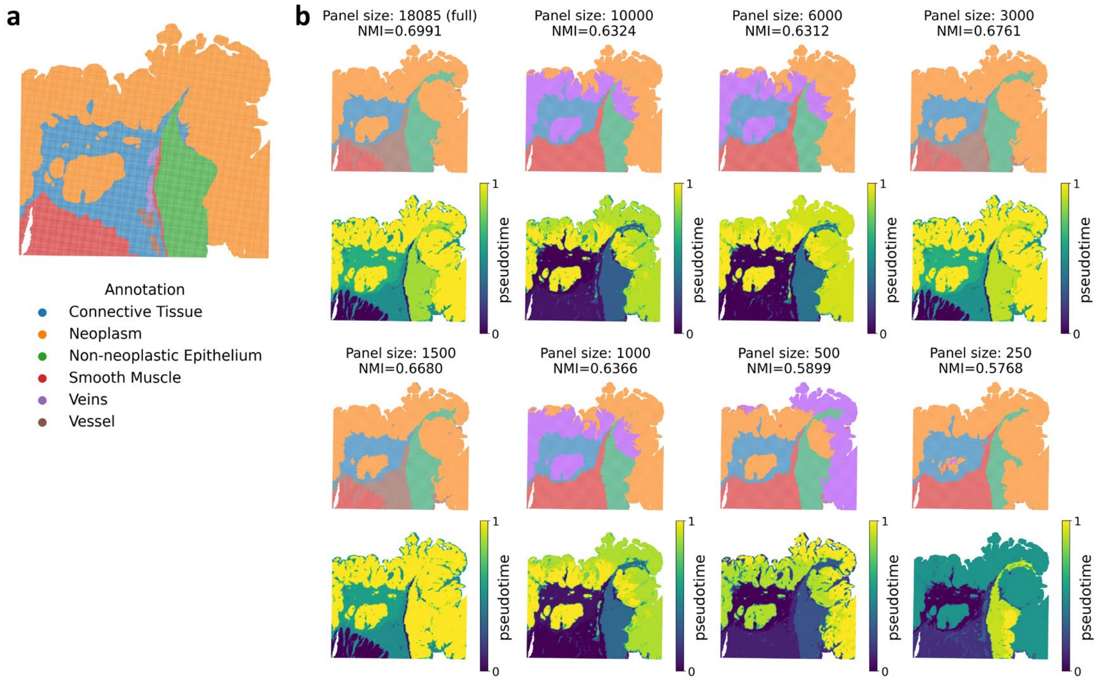

Figure S8. Sensitivity of SECTOR to gene panel size on the Visium HD CRC 16-μm binned output. (**a**) Ground-truth annotation. (**b**) SECTOR outputs after progressively restricting the input feature set to nested gene panels defined by a single global HVG ranking computed from the full transcriptome (18,085 genes). Panel sizes were 18,085, 10,000, 6,000, 3,000, 1,500, 1,000, 500 and 250 genes. For each panel size, the upper panel shows the inferred spatial domains together with NMI against the annotation, and the lower panel shows the inferred pseudotime field. SECTOR remained robust across moderate reductions in panel size, with clustering and pseudotemporal structure largely preserved down to approximately 1,500–3,000 genes, whereas performance and spatial coherence declined more noticeably at 500 and 250 genes.

S8 Ablation analysis of cluster-balance regularisation, early stopping and post hoc refinement

To assess the contribution of SECTOR's optional stabilisation and refinement components, we performed an ablation study on the representative 10x Visium DLPFC slice 151673 (Supplementary Fig. S9). Starting from the default configuration, we disabled one component at a time while keeping other settings unchanged: the cluster-balance penalty, early stopping, or post hoc island cleanup. The default model achieved an NMI of 0.7126 and recovered spatial domains closely aligned with the cortical layer annotations. Removing the cluster-balance penalty reduced NMI to 0.6743 and produced less anatomically consistent domains, indicating that balancing can help maintain stable use of the specified domain structure in this slice. Removing post hoc refinement also reduced NMI to 0.6947 and introduced more local irregularities, consistent with its role in cleaning small isolated fragments. In contrast, disabling early stopping yielded a comparable NMI of 0.7140 in this example, suggesting that early stopping acts mainly as a practical safeguard for training stability and efficiency rather than guaranteeing higher NMI in every run. Overall, the ablation supports the use of these components as optional stabilisation and refinement steps that can improve domain consistency when needed.

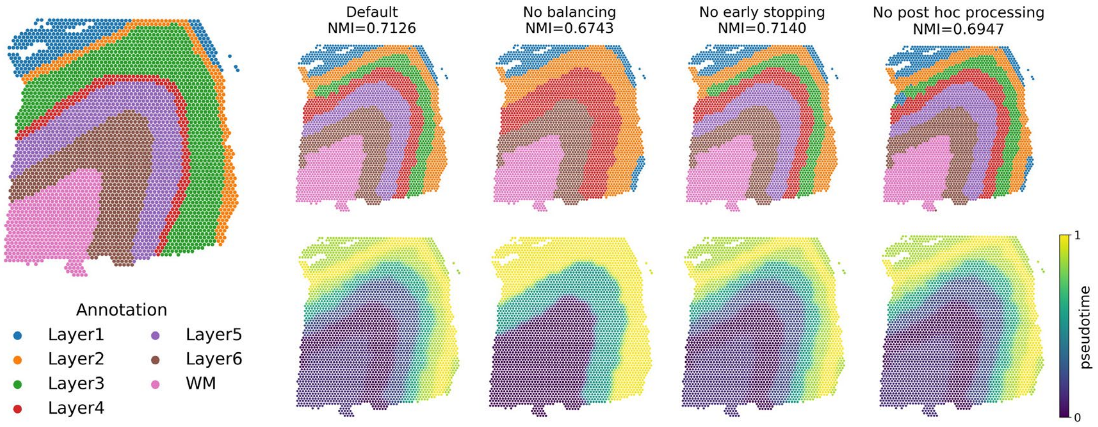

Figure S9. Ablation analysis of SECTOR components on the 10x Visium DLPFC slice 151673. Manual cortical-layer annotations are shown on the left. For each SECTOR variant, the upper panel shows predicted spatial domains and the lower panel shows inferred pseudotime. From left to right: default SECTOR, NMI = 0.7126; without cluster-balance regularisation, NMI = 0.6743; without early stopping, NMI = 0.7140; and without post hoc island cleanup, NMI = 0.6947. The results indicate that cluster-balance regularisation and post hoc refinement can improve spatial-domain stability and anatomical coherence, whereas early stopping mainly provides a practical safeguard for training stability and does not necessarily increase NMI in every individual run.

References

Andersson, A., *et al.* Spatial deconvolution of HER2-positive breast cancer delineates tumor-associated cell type interactions. *Nature Communications* 2021;12(1):6012.

Bhuva, D.D., *et al.* Library size confounds biology in spatial transcriptomics data. *Genome Biology* 2024;25(1):99.

Chen, A., *et al.* Spatiotemporal transcriptomic atlas of mouse organogenesis using DNA nanoball-patterned arrays. *Cell* 2022;185(10):1777-1792.e1721.

Chen, X., *et al.* Efficient in situ barcode sequencing using padlock probe-based BaristaSeq. *Nucleic Acids Research* 2018;46(4):e22-e22.

Cressie, N. and Hawkins, D.M. Robust estimation of the variogram: I. *Journal of the International Association for Mathematical Geology* 1980;12(2):115-125.

Dong, K., *et al.* Benchmarking multi-slice integration and downstream applications in spatial transcriptomics data analysis. *Genome Biology* 2025;26(1):318.

Li, Z. and Zhou, X. BASS: multi-scale and multi-sample analysis enables accurate cell type clustering and spatial domain detection in spatial transcriptomic studies. *Genome Biology* 2022;23(1):168.

Long, B., Miller, J. and Consortium, S. SpaceTx: a roadmap for benchmarking spatial transcriptomics exploration of the brain. *arXiv preprint arXiv:2301.08436* 2023.

Maynard, K.R., *et al.* Transcriptome-scale spatial gene expression in the human dorsolateral prefrontal cortex. *Nature Neuroscience* 2021;24(3):425-436.

Moffitt, J.R., *et al.* Molecular, spatial, and functional single-cell profiling of the hypothalamic preoptic region. *Science* 2018;362(6416):eaau5324.

Oliveira, M.F.d., *et al.* High-definition spatial transcriptomic profiling of immune cell populations in colorectal cancer. *Nature Genetics* 2025;57(6):1512-1523.

Pham, D., *et al.* Robust mapping of spatiotemporal trajectories and cell–cell interactions in healthy and diseased tissues. *Nature Communications* 2023;14(1):7739.

Ren, H., *et al.* Identifying multicellular spatiotemporal organization of cells with SpaceFlow. *Nature Communications* 2022;13(1):4076.

Schaub, D.P., *et al.* PCA-based spatial domain identification with state-of-the-art performance. *Bioinformatics* 2025;41(1).

Shang, L. and Zhou, X. Spatially aware dimension reduction for spatial transcriptomics. *Nature Communications* 2022;13(1):7203.

Sun, J., *et al.* Beyond benchmarking: an expert-guided consensus approach to spatially aware clustering. *bioRxiv* 2025.

Túrós, D., *et al.* Chrysalis: decoding tissue compartments in spatial transcriptomics with archetypal analysis. *Communications Biology* 2024;7(1):1520.

Wang, X., *et al.* Three-dimensional intact-tissue sequencing of single-cell transcriptional states. *Science* 2018;361(6400):eaat5691.

Yu, Y., *et al.* Inferring causal trajectories from spatial transcriptomics using CASCAT. *Nucleic Acids Research* 2025;53(15).

Yuan, Z., *et al.* Benchmarking spatial clustering methods with spatially resolved transcriptomics data. *Nature Methods* 2024;21(4):712-722.

Zhang, J., *et al.* Unsupervised Graph Clustering with Deep Structural Entropy. In, *Proceedings of the 31st ACM SIGKDD Conference on Knowledge Discovery and Data Mining V.2*. Toronto ON, Canada: Association for Computing Machinery; 2025. p. 3752–3763.

Zhang, M., *et al.* Molecularly defined and spatially resolved cell atlas of the whole mouse brain. *Nature* 2023;624(7991):343-354.
